# FoxO1 in dopaminergic neurons regulates energy homeostasis and targets tyrosine hydroxylase

**DOI:** 10.1038/ncomms12733

**Published:** 2016-09-29

**Authors:** Khanh V. Doan, Ann W. Kinyua, Dong Joo Yang, Chang Mann Ko, Sang Hyun Moh, Ko Eun Shong, Hail Kim, Sang-Kyu Park, Dong-Hoon Kim, Inki Kim, Ji-Hye Paik, Ronald A. DePinho, Seul Gi Yoon, Il Yong Kim, Je Kyung Seong, Yun-Hee Choi, Ki Woo Kim

**Affiliations:** 1 Department of Pharmacology, Wonju College of Medicine, Yonsei University, Wonju 26426, Korea; 2 Department of Global Medical Science, Wonju College of Medicine, Yonsei University, Wonju 26426, Korea; 3 School of Pharmacy, University of Medicine and Pharmacy-Ho Chi Minh, 41 Dinh Tien Hoang, District 1, Ho Chi Minh city 700000, Vietnam; 4 Anti-aging Research Institute of BIO-FD&C Co. Ltd., Incheon 21990, Korea; 5 Graduate School of Medical Science and Engineering, Korea Advanced Institute of Science and Technology, Daejeon 34141, Korea; 6 Department of Biochemistry, College of Medicine, Catholic Kwandong University, Gangneung 25601 Korea; 7 Department of Pharmacology, Korea University College of Medicine, Seoul 02841, Korea; 8 Asan Institute for Life Sciences, Asan Medical Center, Seoul 05535, Korea; 9 Department of Pathology and Laboratory Medicine, Weill Cornell Medical College, New York 10065, USA; 10 Department of Cancer Biology, University of Texas MD Anderson Cancer Center, Houston, Texas 77030, USA; 11 Laboratory of Developmental Biology and Genetics, College of Veterinary Medicine, Research Institute for Veterinary Science, Seoul 08826, Korea; 12 Korea Mouse Phenotyping Center, Seoul 08826, Korea; 13 BK21Plus Program for Creative Veterinary Science Research, BIO-MAX institute, Interdisciplinary Program for Bioinformatics, and Program for Cancer Biology, Seoul National University, Seoul 08826, Korea

## Abstract

Dopaminergic (DA) neurons are involved in the integration of neuronal and hormonal signals to regulate food consumption and energy balance. Forkhead transcriptional factor O1 (FoxO1) in the hypothalamus plays a crucial role in mediation of leptin and insulin function. However, the homoeostatic role of FoxO1 in DA system has not been investigated. Here we report that FoxO1 is highly expressed in DA neurons and mice lacking FoxO1 specifically in the DA neurons (FoxO1 KO^DAT^) show markedly increased energy expenditure and interscapular brown adipose tissue (iBAT) thermogenesis accompanied by reduced fat mass and improved glucose/insulin homoeostasis. Moreover, FoxO1 KO^DAT^ mice exhibit an increased sucrose preference in concomitance with higher dopamine and norepinephrine levels. Finally, we found that FoxO1 directly targets and negatively regulates tyrosine hydroxylase (TH) expression, the rate-limiting enzyme of the catecholamine synthesis, delineating a mechanism for the KO phenotypes. Collectively, these results suggest that FoxO1 in DA neurons is an important transcriptional factor that directs the coordinated control of energy balance, thermogenesis and glucose homoeostasis.

## Abstract (web summary)

Dopaminergic neurons are important for regulating energy homeostasis. Here, the authors show the transcription factor FoxO1 negatively regulates tyrosine hydroxylase expression in midbrain dopaminergic neurons, and plays an important role in regulation of glucose homeostasis, energy expenditure, and resistance to diet-induced obesity.

## Graphical abstract

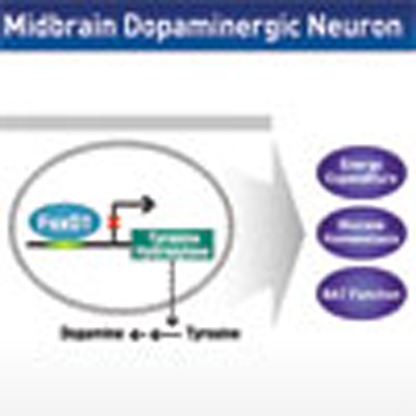

The forkhead transcriptional factor O1, FoxO1, is a main downstream effector of insulin receptor substrate/phosphoinositide 3-kinase/protein kinase B (IRS/PI3K/PKB) pathway and plays an important role in regulation of whole-body metabolism and energy homoeostasis^1^. In the central nervous system (CNS), particularly in the arcuate nucleus (ARC) and the ventral medial nucleus of the hypothalamus (VMH), FoxO1 is a key regulator of insulin/leptin-mediated food intake and energy expenditure^2^,^3^,^4^. In agouti-related peptide (AgRP)- and pro-opiomelanocortin (POMC)-expressing neurons of the ARC region, FoxO1 promotes the expression of AgRP and suppresses the expression of POMC directly or via antagonizing the signal transducer and activator of transcription 3 (STAT3), which consequently leads to increased food intake and body weight^2^,^3^,^5^. In steroidogenic factor 1 (SF-1) neurons within the VMH, FoxO1 suppresses energy expenditure and transcriptionally down-regulates the expression of SF-1 (ref. 4). Recent studies suggested that FoxO1 might function beyond the hypothalamus^6^,^7^,^8^. However, the homoeostatic role of FoxO1 in the neuronal populations of other brain regions remains unknown.

Dopaminergic (DA) neurons are emerging as a critical subset of neurons involved in the integration of neuronal and hormonal signals to regulate food consumption and energy balance^9^,^10^,^11^. Compelling evidence indicates that peripheral metabolic hormones such as insulin and leptin act on their receptors not only in the hypothalamus but also in the DA neurons to regulate feeding and energy intake^10^,^11^. The presence of functional IRS2/PI3K/Akt and Janus kinase (Jak)/STAT3 signalling pathways in DA system^12^,^13^ led us to hypothesize that FoxO1 in the DA neurons might play a critical role in regulating food intake and metabolic homoeostasis.

In the present study, we investigated the functional significance of DA FoxO1 in metabolic homoeostasis using DA neurons specific FoxO1 KO (FoxO1 KO^DAT^) mice. Our results indicate that FoxO1 in DA neurons regulates not only body weight and glucose/insulin homoeostasis, but also plays an important role in energy expenditure and interscapular brown adipose tissue (iBAT) function. Moreover, FoxO1 KO^DAT^ mice exhibit higher sucrose preference in concomitance with increased level of catecholamines (CAs), dopamine and norepinephrine. Finally, we identified tyrosine hydroxylase (TH) as a direct transcriptional target of FoxO1, suggesting a mechanism through which DA FoxO1 regulates whole-body energy homoeostasis.

## Results

### Specific FoxO1 deletion in DA neurons

**Figure 1:**
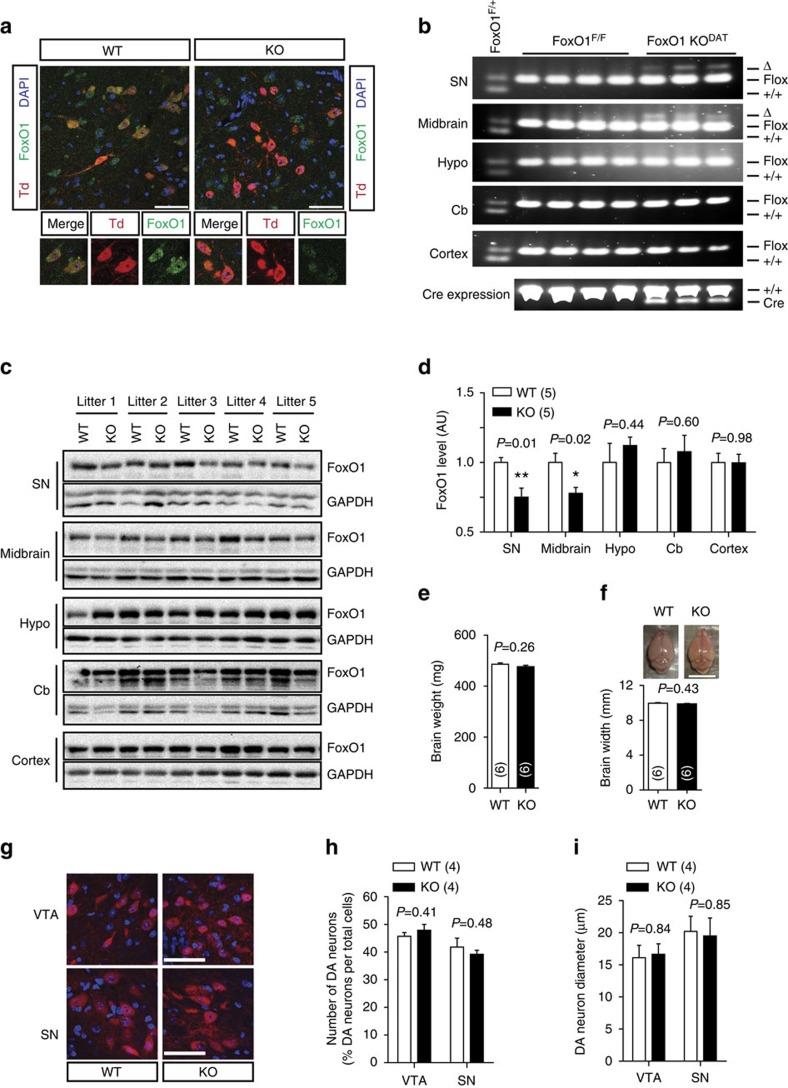
Generation of DA neuron-specific FoxO1 KO (FoxO1 KO^DAT^) mice. (**a**) DA neuron-specific FoxO1 deletion. Green fluorescence indicates FoxO1. TdTomato (Td, red) was used to visualize DA neurons expressing DAT-cre. DAPI stains nuclei (blue). Scale bar, 50 μm. (**b**) Allele-specific PCR using different brain areas from FoxO1^F/+^, FoxO1^F/F^, and FoxO1 KO^DAT^ (DAT-cre; FoxO1^F/F^). SN, substantia nigra. Hypo., hypothalamus. Cb, cerebellum. (**c**,**d**) Immunoblots (**c**) and relative FoxO1 protein levels (**d**) from indicated brain regions of FoxO1 KO^DAT^ and WT littermates. (**e**) Brain weight of FoxO1 KO^DAT^ and WT littermates. (**f**) Representative figures and graphs showing the brain width of FoxO1 KO^DAT^ and WT littermates. Scale bar, 10 mm. (**g**–**i**) Representative figures (**g**) and graphs showing the number (**h**) and size (**i**) of DA neurons of FoxO1 KO^DAT^ and WT littermates. Scale bar, 50 μm. The values are mean±s.e.m. (*P<0.05, **P<0.01, Student's *t*-test).

To directly determine the role of FoxO1 in DA neurons-directed metabolic homoeostasis, we generated DA-specific FoxO1 knockout mice (KO, FoxO1 KO^DAT^) by crossing DAT^IREScre^ with FoxO1^*loxP/loxP*^ (FoxO1^F/F^) mice^4^,^14^,^15^. Littermate mice homozygous for the floxed FoxO1 allele (FoxO1^F/F^) and without the DAT^IREScre^ allele served as controls (WT, wild-type). The tdTomato reporter mice^16^ and co-immunostaining with antibody against TH-verified DAT^IREScre^ activity specifically in DA neurons as reported (Supplementary Fig. 1a–c)^15^,^17^. Immunofluorescence showed robust FoxO1 signal in virtually all DA neurons and the elimination of that signal in the KO mice (Fig. 1a). Furthermore, allele-specific PCR confirmed FoxO1 locus rearrangement (delta band), consistent with the deletion of FoxO1 in DA neurons including substantia nigra (SN) and midbrain containing the ventral tegmental area (VTA)^18^ but not in other brain regions such as hypothalamus, cortex, cerebellum and peripheral tissues including adrenal glands, BATs, muscles, livers or in WT mice (Fig. 1b and Supplementary Fig. 1d). In addition, FoxO1 protein levels of the KO mice were significantly decreased in VTA and SN but not in other CNS areas (Fig. 1c,d, and Supplementary Fig. 7) or peripheral tissues (Supplementary Fig. 1e,f, and Supplementary Fig. 7). Deletion of FoxO1 in DA neurons did not affect the brain size, the number and size of DA neurons (Fig. 1e-i). Taken together, these results confirm that FoxO1 was successfully and specifically deleted in DA neurons without effect on normal brain development and the FoxO1 KO^DAT^ mice provide a model to investigate potential metabolic roles of FoxO1 in DA neurons *in vivo*.

### Improved glucose/insulin homoeostasis in FoxO1 KO^DAT^ mice

**Figure 2:**
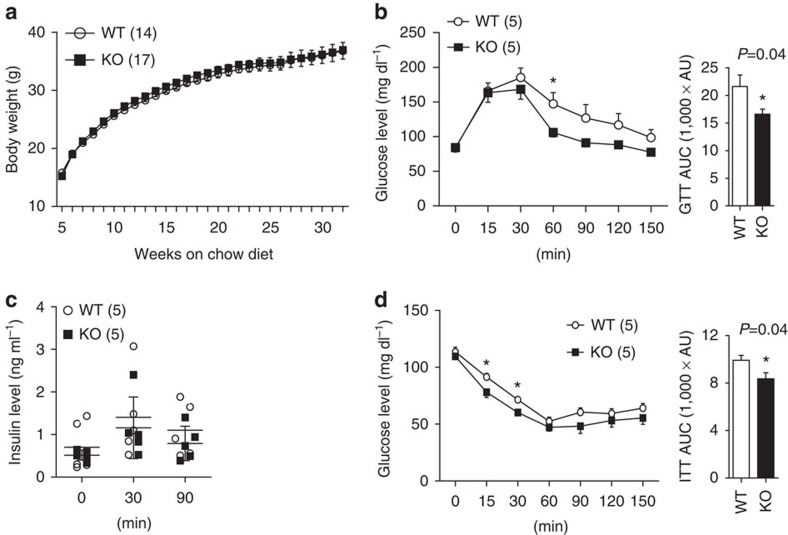
Metabolic phenotypes of FoxO1 KO^DAT^ mice in chow diet. (**a**) Body weight of male mice on chow diet. Body weight data were combined from three cohorts of mice. (**b**) GTT and area under curve (AUC) of 24-week old male mice fed chow diet. (**c**) Plasma insulin levels of male mice during GTT (Not significant from two-way analysis of variance (ANOVA)). (**d**) ITT and AUC of 24-week-old male mice on chow diet. The results are expressed as mean±s.e.m. (*P<0.05, Student's *t*-test for bar graphs and two-way ANOVA for comparison of multiple time points).

In chow diet, body weight and growth of KO mice was comparable to WT littermates in both sexes (Fig. 2a and Supplementary Fig. 2a). Similarly, there was no observable difference in fed and fasted glucose levels and serum insulin and leptin concentrations (Supplementary Fig. 2b–i). However, unexpectedly, the KO mice exhibited modestly but significantly improved glucose and insulin sensitivity with no difference in insulin levels when the mice were challenged with glucose and insulin (Fig. 2b–d and Supplementary Fig. 2j–k). These results show that deletion of FoxO1 in DA neurons enhanced glucose/insulin sensitivity without affecting the insulin secretion.

### Resistance to diet-induced obesity in FoxO1 KO^DAT^ mice

**Figure 3:**
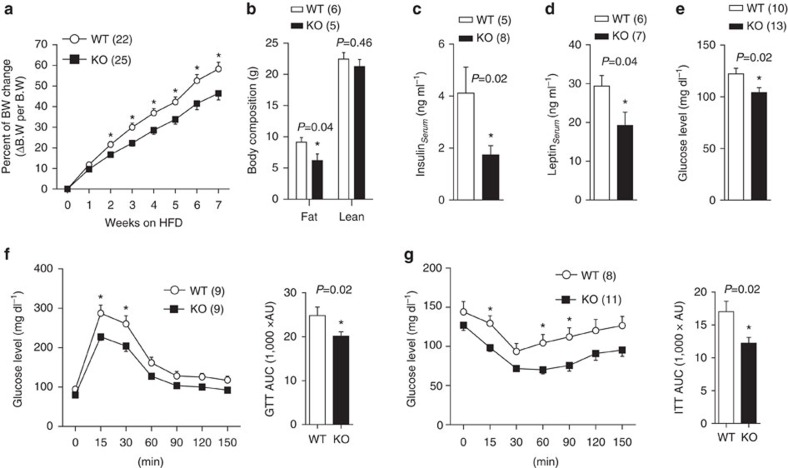
FoxO1 KO^DAT^ mice are resistant to diet-induced obesity. (**a**) Body weight change (%) after HFD feeding (HFD started at 8 weeks old). Body weight data were combined from 4 cohorts. (**b**) Body fat and lean mass of male mice at 6 weeks on HFD. (**c**,**d**) Serum insulin (**c**) and leptin (**d**) levels of male mice at 6 weeks on HFD (Body weight, WT: 38.93±1.75 g, KO: 34.95±1.63 g, *P*=0.13 for **c** and WT: 37.87±1.78 g, KO: 33.92±1.50 g, *P*=0.12 for **d**, respectively). (**e**) Blood glucose (fed) level of male mice at 10 weeks on HFD (Body weight, WT: 42.71±2.12 g and KO: 36.18±2.17 g, *P*=0.04). (**f**) GTT and AUC of male mice on HFD for 10 weeks (Body weight, WT: 37.94±2.07 g and KO: 32.74±1.31 g, *P*=0.04). (**g**) ITT and AUC of male mice on HFD for 10 weeks (Body weight, WT: 39.64±1.96 g and KO: 33.86±1.55 g, *P*=0.03). The results are expressed as mean±s.e.m. (*P<0.05, Student's *t*-test for bar graphs and two-way analysis of variance for comparison of multiple time points in line graphs).

Prompted by metabolic phenotypes of FoxO1 KO^DAT^ mice seen only in stress conditions such as glucose and insulin tolerance tests (GTT and ITT), we next challenged the mice with high-fat diet (HFD) to investigate the role of FoxO1 in DA neurons in metabolic stress condition. Interestingly, FoxO1 KO^DAT^ mice on HFD showed significantly less weight gain compared to WT littermates (Fig. 3a and Supplementary Fig. 2l). Less weight gain under HFD in FoxO1 KO^DAT^ mice was associated with reduced total fat mass (Fig. 3b). Furthermore, HFD-fed FoxO1 KO^DAT^ mice showed significantly decreased blood glucose, insulin and leptin levels (Fig. 3c–e). In addition, GTT and ITT revealed an enhanced glucose and insulin sensitivity in FoxO1 KO^DAT^ mice compared to their WT littermates (Fig. 3f,g and Supplementary Fig. 2m). These results illustrate that FoxO1 in DA neurons plays an important role not only in glucose/insulin homoeostasis but also in body weight regulation, especially under HFD condition.

### Food intake and feeding behaviour in FoxO1 KO^DAT^ mice

**Figure 4:**
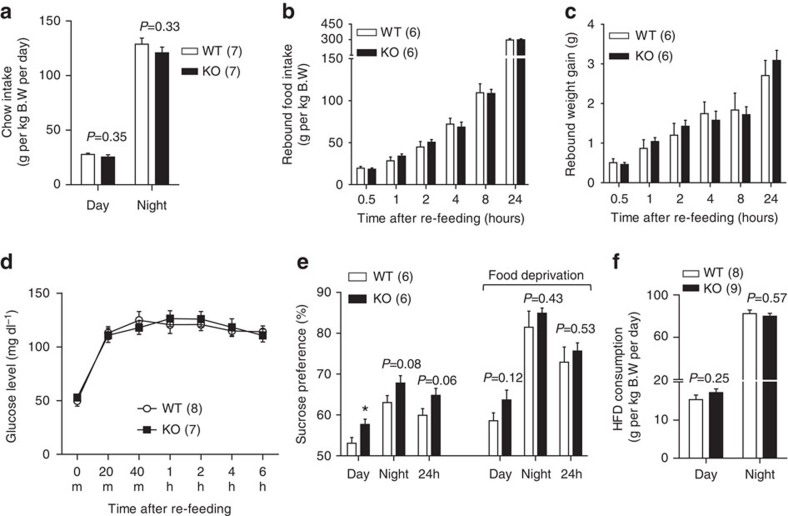
Feeding behaviour of FoxO1 KO^DAT^ mice. (**a**) Daily food intake of 8-10 weeks old male mice on chow diet (averaged from 7 days). (**b**–**d**) Rebound food intake (**b**) rebound weight gain (**c**) and rebound blood glucose (**d**) of overnight fasted male mice after re-feeding with normal chow. (**e**) Percentage of sucrose preference in fed and fasted conditions of male mice. 2% sucrose was used for the preference test. (**f**) Daily HFD consumption of 8-10 weeks old male mice (averaged from 7 days). The results are expressed as mean±s.e.m. (*P<0.05, Student's *t*-test for bar graphs in **a**,**e**–**f** and two-way analysis of variance for comparison of multiple time points in **b**–**d**).

DA neurons are involved in regulation of food consumption by modulating feeding and food reward^19^ and previous studies have reported the importance of FoxO1 in the control of food intake^2^,^3^,^20^,^21^. Therefore, we postulated that the improved glucose/insulin sensitivity and resistance to diet-induced obesity in FoxO1 KO^DAT^ mice were due, at least in part, to decreased food intake. However, FoxO1 KO^DAT^ mice showed no difference in food intake compared with WT mice in chow diet (Fig. 4a). Next, we examined whether FoxO1 deletion in DA neurons has effect on fasting and re-feeding response^4^,^6^. Re-feeding experiments in chow diet, however, failed to detect any changes in rebound food intake, weight gain or blood glucose between WT and FoxO1 KO^DAT^ mice (Fig. 4b–d and Supplementary Fig. 3a and b). Accordingly, there was no difference in the expression of hypothalamic neuropeptides involved in feeding behaviour including *Agrp*, *Npy*, *Pomc* and *Cart* (Supplementary Fig. 3c).

The regulation of reward feeding or palatable food consumption by DA neurons has been well studied^10^. Moreover, central deletion of FoxO1 has been shown to enhance anorexigenic effect^2^,^3^,^20^,^21^,^22^. This led us to investigate whether FoxO1 in DA neurons is involved in the regulation of reward-related feeding and palatable food consumption. To address this question, sucrose preference tests were performed in FoxO1 KO^DAT^ mice. While 1 and 5% sucrose showed no difference between genotypes (Supplementary Fig. 3d), FoxO1 KO^DAT^ mice showed increased sucrose intake in 2% sucrose compared with WT littermates, which was not evident in fasted condition (Fig. 4e). These results indicated that FoxO1 in DA neurons seems to be involved in the regulation of specific feeding behaviours such as palatable food consumption. We next examined the contribution of food reward aspect to HFD consumption in the KO mice. However, FoxO1 KO^DAT^ mice showed no difference in HFD intake compared with WT littermates (Fig. 4f). Taken together, these results suggest that the difference in reward-related feeding in FoxO1 KO^DAT^ mice might not affect the homoeostatic feeding as the total calories intake was unchanged (Fig. 4a,f and Supplementary Fig. 3e), indicating that energy intake might not be the reason for the observed phenotypes in FoxO1 KO^DAT^ mice.

### Increased energy expenditure in FoxO1 KO^DAT^ mice

**Figure 5:**
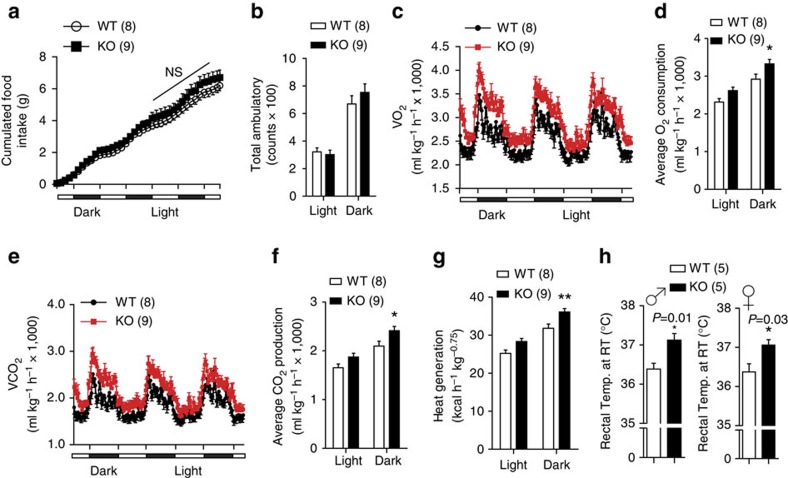
Increased energy expenditure in FoxO1 KO^DAT^ mice. (**a**) Cumulative food intake of male mice fed on HFD for 1 week. (**b**) Locomotor activity. (**c**) Temporal changes of O_2_ consumption. (**d**) Average O_2_ consumption. (**e**) Temporal changes of CO_2_ production. (**f**) Average CO_2_ production. (**g**) Heat generation between genotypes. (**h**) Rectal temperature of male and female mice measured at room temperature environment. ♂, male. ♀, female. NS, not significant. Data are expressed as mean±s.e.m. (*P<0.05, Student's *t*-test for bar graphs in **h** and two-way analysis of variance for multiple comparisons in **a**–**g**).

To obtain mechanistic insights into the improved metabolic profiles and to assess the cause of lower body weight in FoxO1 KO^DAT^ mice especially under HFD challenge, we analysed food intake, energy expenditure and physical activity of the HFD-fed mice using metabolic chambers. To rule out secondary metabolic effects of body weight difference, mice fed on HFD for 1 week with comparable body weight were used (Supplementary Fig. 2l). Although there was a minor trend towards an increase in HFD intake, the FoxO1 KO^DAT^ mice showed no significant difference in total accumulated food intake (Fig. 5a). In addition, physical activity of FoxO1 KO^DAT^ mice (either inside or outside of the metabolic chambers) was similar to WT controls (Fig. 5b and Supplementary Fig. 4a). In contrast, measurements of respiratory exchange demonstrated an increase in O_2_ consumption and CO_2_ production in KO mice (Fig. 5c–f). Moreover, FoxO1 KO^DAT^ mice showed robustly increased heat generation compared with WT littermates (Fig. 5g). In line with the increased energy expenditure, the rectal temperature of KO mice was higher than that of WT controls (Fig. 5h). These results strongly indicate that increased energy expenditure might be a major cause of the metabolic phenotypes observed in FoxO1 KO^DAT^ mice.

### CAs level and iBAT thermogenesis in FoxO1 KO^DAT^ mice

**Figure 6:**
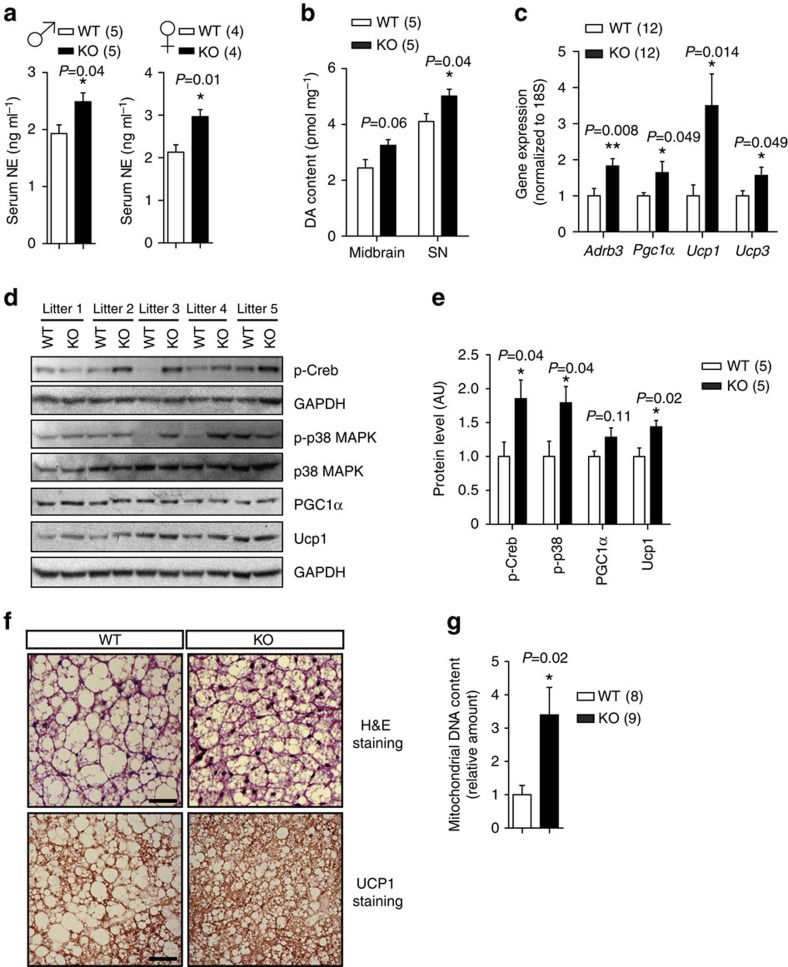
Increased catecholamines level and iBAT thermogenesis in FoxO1 KO^DAT^ mice. (**a**) Serum norepinephrine levels of WT and KO male and female mice. (**b**) Dopamine contents measured from the midbrain and SN samples of WT and KO male mice. (**c**) Gene expression in iBAT of WT and KO male mice. (**d**) Immunoblots for p-Creb, p-p38 MAPK, UCP1, PGC1α in iBAT. (**e**) Relative protein levels for p-Creb, p-p38 MAPK, UCP1, PGC1α in iBAT from (**d**). Normalized to total p38 MAPK or GAPDH. (**f**) Representative figures of H&E staining and UCP1 staining from iBAT samples of WT and KO mice (*n*=3). Scale bar, 50 μm. (**g**) Quantification of mitochondrial DNA contents from iBAT samples of WT and KO mice. ♂, male. ♀, female. Data are expressed as mean±s.e.m. (*P<0.05, **P<0.01, Student's *t*-test).

With no difference in physical activity and food intake, we assumed that enhanced thermogenesis might contribute to the increased energy expenditure in FoxO1 KO^DAT^ mice. Since sympathetic nervous system plays a crucial role in control of adaptive thermogenesis^23^, we first checked serum level of norepinephrine in FoxO1 KO^DAT^ mice. As expected, serum norepinephrine level of KO mice was significantly elevated suggesting the increment of sympathetic nervous system in FoxO1 KO^DAT^ (Fig. 6a and Supplementary Fig. 4b). Interestingly, we also observed an elevation in dopamine contents in midbrain and SN of the KO mice (Fig. 6b).

It is known that increased sympathetic flow from the CNS to iBAT plays an important role in the activation of thermogenesis^24^,^25^ and the central DA activity in addition is involved in the regulation of iBAT thermogenesis^26^,^27^. Therefore, we wondered whether FoxO1 ablation in DA neurons would have any effects on iBAT thermogenesis. Indeed, the genes regulating iBAT thermogenesis including *Adrb3, Pgc1α, Ucp1* and *Ucp3* were significantly increased in KO mice (Fig. 6c). Consistent with increased level of CAs, iBAT of KO mice showed significantly increased levels of Ucp1 and phosphorylation of Creb and p38 MAPK which are downstream targets of CA/Adrb3 signalling^28^ (Fig. 6d,e and Supplementary Fig. 7). In addition, iBATs of KO mice showed increased UCP1 staining, mitochondrial DNA contents and less lipid accumulation (Fig. 6f,g). These data suggest that increased sympathetic activity might contribute to the increased energy expenditure and resistance to diet-induced obesity phenotype in FoxO1 KO^DAT^ mice.

### D2R signalling and DA turnover in FoxO1 KO^DAT^ mice

The increase in dopamine contents in the midbrain led us to ask whether there is any change in dopamine receptor 2 (D2R) signalling. Interestingly, we observed modest reduction in D2R expression in the midbrain of the FoxO1 KO^DAT^ mice (Supplementary Fig. 5a and b). Although the relationship between obesity and D2R receptor availability is still controversial, altered D2R receptor availability has been implicated in over-eating and obesity development^29^,^30^,^31^,^32^,^33^,^34^. However, we did not observe any difference in total food intake (both normal chow (NC) and HFD) in the FoxO1 KO^DAT^ mice even though those mice exhibited modestly increased sucrose preference and increasing trend in the HFD consumption during metabolic chamber analyses (Figs 4,5a). Moreover, there was no difference in physical activity (Fig. 5b and Supplementary Fig. 4a). It is likely that decreased D2R expression in the KO mice was not a primary effect of DA FoxO1 deletion, but a secondary effect due to compensatory feedback regulation from the increase in dopamine content.

Recent studies suggest that insulin resistance in obesity is linked to changes in DA turnover^35^,^36^. We, therefore, explored DA turnover in FoxO1 KO^DAT^ mice by examining the expression of enzymes, catechol-O-methyltransferase (COMT) and monoamine oxidase b (MAO-b), which catalyse the conversion of dopamine into 3-methoxytyramine (3-MT) and 3,4-dihydroxyphenylacetic acid (DOPAC), respectively. However, FoxO1 KO^DAT^ mice showed comparable COMT and MAO-b expression suggesting that the DA turnover might not be changed in KO mice (Supplementary Fig. 5a–d).

### Prolactin level in FoxO1 KO^DAT^ mice

The small population of DA neurons in the hypothalamus has been known to regulate the secretion of prolactin which also plays a role in regulating food intake and energy balance^37^,^38^. We, therefore, examined whether deletion of FoxO1 in DA neurons affects prolactin secretion from the pituitary. However, there was no detectable change in prolactin levels in the pituitary and in the serum samples of FoxO1 KO^DAT^ mice (Supplementary Fig. 5e and f). Consistent with unaltered DA turnover and hypothalamic TH expression (Supplementary Fig. 5a–d and Supplementary Fig. 6a–c), unchanged prolactin level was in line with the aforementioned observation that FoxO1 KO^DAT^ mice showed no difference in homoeostatic feeding and food intake. Although these results could not totally exclude the possibility of an alteration in the hypothalamic-pituitary system caused by FoxO1 deletion in DA neurons of the hypothalamus, unchanged prolactin levels implied that the metabolic phenotypes observed in KO mice might not be the secondary effects of prolactin secretion inhibition.

### FoxO1 directly regulates TH expression

**Figure 7:**
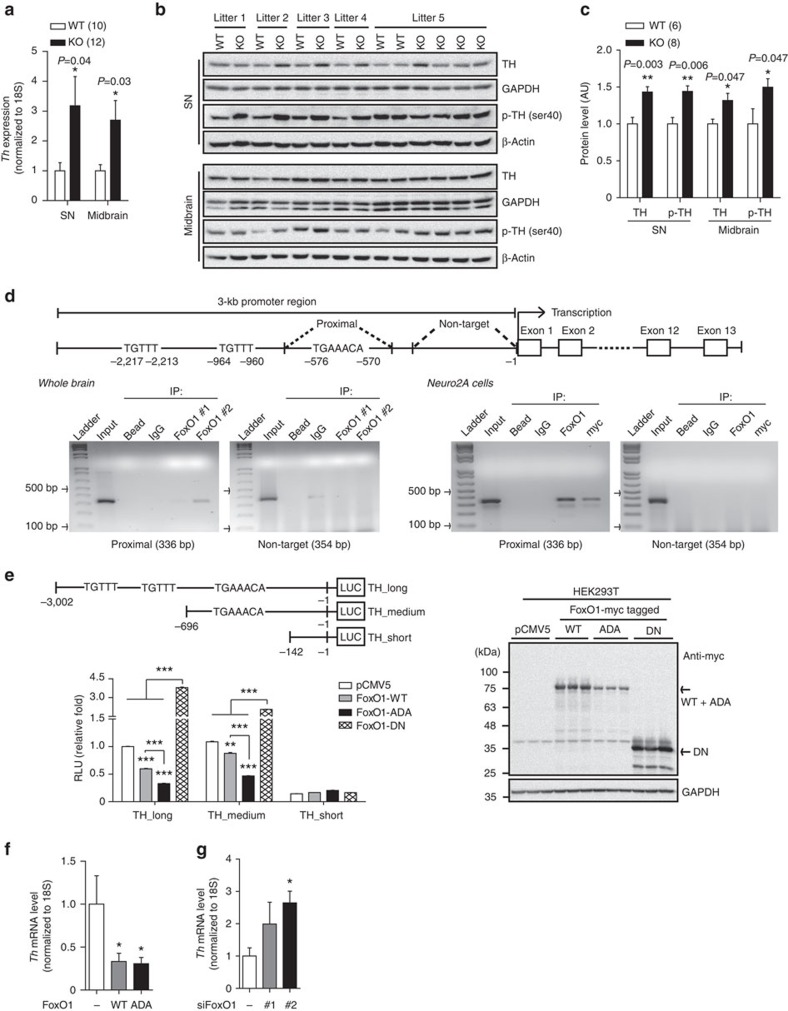
FoxO1 directly regulates tyrosine hydroxylase (TH) expression in DA neurons. (**a**) mRNA level of *Th* in DA neurons of WT and KO littermates. (**b**) Immunoblots for TH and phosphorylated TH (p-TH) in SN and midbrain from WT and KO littermates. (**c**) Relative TH and p-TH protein levels from **b**. (**d**) Top, schematic diagram for mouse TH promoter. Bottom, ChIP assays using whole-brain and Neuro2A cells transfected with myc-tagged FoxO1-ADA showing a direct and specific binding of FoxO1 on the proximal region of TH promoter. (**e**) Top, schematic diagram for luciferase constructs with or without FoxO1 potential binding sites. Bottom left, relative luciferase activity after FoxO1-WT (WT), constitutive active form of FoxO1 (ADA) and dominant-negative form of FoxO1 (DN) overexpression (*n*=6). Bottom right, immunoblots confirming expression of FoxO1-WT, -ADA, and -DN. (**f**) *Th* mRNA expression in Neuro2A cells after FoxO1-WT and -ADA overexpression (*n*=9). (**g**) *Th* mRNA level in Neuro2A cells after FoxO1 knockdown (*n*=9). The results are expressed as mean±s.e.m. (*P<0.05, **P<0.01, ***P<0.001, Student's *t*-test and one-way analysis of variance for luciferase and quantitative real-time PCR analyses) from more than three independent experiments.

Increased dopamine and norepinephrine levels with no difference in DA turnover in KO mice led us to hypothesize that FoxO1 is directly involved in CA synthesis in DA neurons. We found a significant increase in mRNA and protein levels of TH in DA neurons of KO mice, which was not detected in other brain regions (Fig. 7a–c and Supplementary Fig. 6a–c). Moreover, the phosphorylated form of TH was significantly increased in DA neuron regions of KO mice further suggesting the increase in TH activity and CA synthesis in KO mice (Fig. 7a–c and Supplementary Fig. 6a–c). Since the highly elevated TH expression was accompanied by an increase in dopamine and norepinephrine levels in KO mice, we speculated that FoxO1 might transcriptionally regulate TH expression. Indeed, sequence analysis revealed three potential FoxO1-binding sites including one conserved FoxO1-binding motif at the proximal region and two insulin-responsive elements (IREs) at the middle and distal regions within −3 kb of TH promoter (Fig. 7d)^39^. We therefore performed chromatin immonoprecipitaion (ChIP) assays to determine whether FoxO1 binds directly to these potential binding sites. Both *in vivo* and *in vitro* ChIP assays using whole-brain and Neuro2A cells transfected with myc-tagged FoxO1-ADA, confirmed a specific and direct binding of FoxO1 on the conserved FoxO1-binding sequence at the proximal region of TH promoter (Fig. 7d). The binding of FoxO1 on the IREs in the middle and distal regions of TH promoter might not be specific as the ChIP signals were also observed with the bead and negative IgG control (Supplementary Fig. 6d). Based on the ChIP analyses, we next established luciferase constructs containing TH promoter regions with or without FoxO1 potential binding sites and measured the promoter activity (Fig. 7e). Overexpression of FoxO1 (WT FoxO1) significantly suppressed the luciferase activity of the constructs containing the potential binding sites and this suppression of FoxO1 on TH promoter was more pronounced in constitutive active FoxO1 (ADA FoxO1; Fig. 7e). In contrast, the promoter activity of TH gene was significantly increased after suppression of endogenous FoxO1 by overexpression of dominant-negative form of FoxO1 (DN FoxO1) or by FoxO1 knockdown with small interfering RNAs (siRNAs; Fig. 7e and Supplementary Fig. 6e and Supplementary Fig. 7). The effects of FoxO1s overexpression were not seen with the luciferase construct that does not contain FoxO1-binding site (Fig. 7e) indicating the specific binding of FoxO1 on the TH promoter. In addition, endogenous *Th* expression^40^ was markedly inhibited by the expression of WT and ADA FoxO1 (Fig. 7f and Supplementary Fig. 6f and Supplementary Fig. 7). Conversely, FoxO1 knockdown promoted endogenous *Th* expression (Fig. 7g and Supplementary Fig. 6g and Supplementary Fig. 7). Altogether, these results indicate that FoxO1 directly regulates TH expression both *in vitro* and *in vivo* and therefore might affect CA synthesis in DA neurons (Supplementary Fig. 6h).

## Discussion

Although the metabolic function of FoxO1 has been well-established in specific subsets of neurons in the hypothalamus, particularly in the AgRP and POMC neurons of the ARC^5^, recent studies have suggested that the function of FoxO1 in the CNS might extend beyond the hypothalamus. Heinrich *et al*^8^. reported that deletion of FoxO1 in majority of hypothalamic neurons using transgenic mice expressing Nkx2.1-cre resulted in normal food intake and energy expenditure and did not protect the mice from diet-induced obesity suggesting the heterogeneity of FoxO1 function within the hypothalamus. However, using synapsin-Cre to ablate FoxO1 in broad neuronal populations with minimal effect on AgRP and POMC neurons, Ren *et al*^6^. found that *Syn-*FoxO1 KO mice exhibited a catabolic energy metabolism phenotype associated with increased locomotor activity and blunted re-feeding response due to enhanced sensitivity to hormonal and nutritional signalling pathway in the CNS. Moreover, *Syn-*FoxO1 KO mice showed increased energy expenditure and resistance to HFD-induced obesity^6^. These combined data indicate the metabolic role of FoxO1 in other neuronal populations outside of the hypothalamus.

The relevance of DA system in regulation of feeding and energy consumption led us to focus on the metabolic function of FoxO1 in DA neurons. To address this question, we took advantage of DAT-Cre which is homogenous and specifically expressed in DA neurons^17^ to generate the DA neuron-specific FoxO1 KO mice. Similar to *Syn-*FoxO1 KO mice, the FoxO1^DAT^ KO mice showed increased energy expenditure, improved glucose homoeostasis and protection from HFD-induced weight gain although the FoxO1^DAT^ KO mice displayed normal re-feeding response and physical activity^6^.

Interestingly, FoxO1^DAT^ KO mice displayed higher sucrose preference and elevated tissue dopamine content in the midbrain which is known to modulate the reward value for nutrients^41^,^42^. In this regard, the minor trend towards an increase in accumulated HFD intake in KO mice (Fig. 5a) could be attributed to either higher reward value of palatable food or compensatory feedback regulation of homoeostasis from markedly increased energy expenditure in KO mice or both. Nonetheless, the difference in reward feeding is unlikely to explain the observed metabolic phenotypes of FoxO1^DAT^ KO mice as the total food intake both in chow diet and HFD was comparable. In support of this concept, the expression of hypothalamic neuropeptides involved in feeding behaviour including *Agrp*, *Npy*, *Pomc* and *Cart* was also not changed in FoxO1^DAT^ KO mice.

Remarkably, the FoxO1^DAT^ KO mice showed markedly increased energy expenditure which did not result from increased physical activity but was due to increased adaptive thermogenesis. Enhanced iBAT thermogenesis in the FoxO1^DAT^ KO mice might be attributed to the activation of sympathetic nervous system activity as the level of norepinephrine was elevated in FoxO1^DAT^ KO mice. In addition, increased dopamine content might also contribute to higher iBAT thermogenesis in FoxO1^DAT^ KO mice since pharmacological studies using dopamine reuptake inhibitors have been shown to activate iBAT thermogenesis^26^,^27^. Increased adaptive thermogenesis due to enhanced sympathetic activity from CNS was supported by the findings that expression of *Adrb3* and downstream signalling of CA/Adrb3 including p-Creb, p-p38 MAPK, Ucp1 was markedly increased in iBAT of FoxO1^DAT^ KO mice.

BAT activation has been shown to improve whole-body glucose homoeostasis and insulin sensitivity^43^,^44^,^45^. By oxidizing metabolic substrates into heat instead of chemical energy, BAT plays an important role in clearance of free fatty acids, triglycerides and glucose as well^43^,^46^,^47^. However, while triglycerides and fatty acids are the major substrates for the oxidative metabolism in BAT, glucose only acts as a minor direct substrate^43^,^48^. Moreover, on stimulation, BAT first consumes the intracellular energy stores before using the plasma-borne substrates^43^,^47^. Therefore, short-time BAT activation only minimally contributes to the whole-body plasma glucose utilization^47^. However, plasma glucose oxidation is enhanced in BAT under prolonged activation condition and accounts for up to 30% of the increase in resting energy expenditure^45^. Consistent with these findings, the blood glucose levels of FoxO1 KO^DAT^ mice were not different under NC-fed condition but were significantly lower after chronic exposure to HFD. However, FoxO1 KO^DAT^ mice showed improved glucose tolerance both in NC and HFD compared with WT mice. These results imply that mild activation of BAT in the FoxO1 KO^DAT^ mice under NC diet or acute HFD exposure might primarily enhance lipid oxidative metabolism, which eventually led to improved whole-body glucose homoeostasis. However, prolonged activation of BAT under chronic HFD exposure might also contribute to glucose clearance and result in further improved glucose tolerance and body weight in the FoxO1 KO^DAT^ mice. Subsequently, the improved glucose metabolism in FoxO1 KO^DAT^ mice might be also attributed to the decreased body weight under HFD condition. Altogether, these data strongly indicate that increased thermogenesis and energy expenditure might be the major cause of the metabolic phenotypes observed in FoxO1^DAT^ KO mice.

An advanced finding of the present work is the specification of DA neurons as a target of metabolic actions of FoxO1 in the CNS. As mentioned above, the DAT-cre activity is homogeneous and limited in DA neurons^15^,^17^. Therefore, the observed phenotypes in FoxO1^DAT^ KO mice could be attributed specifically to the DA neurons. In this regard, our data provide strong evidence that FoxO1 in DA neurons not only plays a role in the control of feeding behaviour but is also critical for the regulation of energy expenditure and metabolic homoeostasis. The DA neuron system is considered part of the reward system regulating feeding behaviour especially those related to reward value of food, and is associated with over-eating and obesity development^19^,^49^. However, recent findings suggest that midbrain DA neurons are not homogenous. They receive neuronal inputs from diverse sources and project to different output sites as well^50^,^51^. It would be interesting to investigate whether FoxO1 acts on a specific subset of DA neurons to regulate energy expenditure while other subsets of DA neurons contribute to the food reward modulation. This, together with the precise brain site of DA neurons where FoxO1 mediates the aforementioned metabolic phenotypes, warrants further studies.

Another novel finding of the current study is the identification of transcriptional regulation of TH, the rate-limiting enzyme of the CA synthesis, by FoxO1. By binding directly to the promoter region of *TH* gene, FoxO1 might inhibit the transcription of *TH* gene, resulting in decreased TH expression. Consistent with the results from ChIP and luciferase analyses, FoxO1^DAT^ KO mice showed significant increase in mRNA and protein levels of TH, which might, at least in part, drive the elevation of DA content in DA neurons. Since DA and noradrenergic neurons have closely reciprocal interactions^52^,^53^,^54^,^55^, elevated dopamine content might lead to increased norepinephrine level^52^ and hence together enhance sympathetic nervous system activity^56^. Many factors have been known to be involved in the control of TH transcription including the orphan nuclear receptor Nurr1^57^,^58^, transcription factors related to cAMP-protein kinase A (PKA) signalling^59^, and the heterogeneous nuclear ribonucleoprotein K (hnRNP-K)^60^. Examining whether FoxO1 is involved in the interaction or regulation with these factors in control of TH expression might be another area for future study.

In conclusion, our current study identifies FoxO1 in DA neurons as a key transcriptional factor not only for the regulation of energy and glucose/insulin homoeostasis but also for TH expression and sympathetic activity.

## Methods

### Mice

All animal procedures were approved by the Institutional Animal Care and Use Committee (IACUC) at Wonju College of Medicine, Yonsei University. Mice were kept in controlled room temperature (22-24 °C) with a 12-h light/dark cycle (light on/off at 06:00/18:00 hours) with NC (Research Diets, Cat.No. 7001; 4.25% kcal from fat, 3.82 kcal g^−1^) or HFD (Research Diets, Cat.No. D12492, 60% fat, 5.24 kcal g^−1^).

### Midbrain and SN dissection

For midbrain and SN dissection, the coronal section of the midbrain was selectively cut from the whole-brain. Under the low power dissecting microscope, the areas of cortex, bilateral SN and midbrain containing the VTA were dissected and harvested. All of the brain samples were snap-frozen on dry ice and stored at −80 °C for further analyses^61^.

### Body weight and composition

The body weight of WT and FoxO1 KO^DAT^ mice (male and female) was monitored weekly from the weaning time (5 weeks old). For HFD study, WT and FoxO1 KO^DAT^ littermate, were maintained on regular chow diet until they were 8 weeks old and then switched to HFD for an additional 6–12 weeks. The body composition of WT and FoxO1 KO^DAT^ mice was analysed by nuclear magnetic resonance (LF90 Minispec, Bruker Corp., TX, USA).

### Food intake and re-feeding experiments

Male mice (8–10 weeks old, body weight-matched) were housed individually and provided food (NC or HFD) and water *ad libitum*. The food intake was daily recorded at 8:00 and 17:00 hours during a 7-day period and was normalized to initial body weight. For the re-feeding experiments, mice were fasted overnight for 18 h with water provided *ad libitum*. The re-feeding food intake, body weight and blood glucose were recorded at the indicated time points.

### Locomotor activity

The open field test was performed in a 60 × 60 × 20 cm (length × width × height) chamber. After 2-h acclimation, the activity of mice was recorded (10 min each mouse). Data were acquired and analysed by the video-tracking SMART system version 3.0 (Panlab, Harvard Apparatus, MA, USA).

### Metabolic analysis

WT and KO male littermate mice fed on HFD for 1 week (body weight, WT, 28.64±1.87 g, *n*=8; KO, 27.98±1.56 g, *n*=9; *P*>0.7) were used. To measure metabolic rate, the mice were housed individually in a combined indirect calorimetry system (CaloSys Calorimetry System, TSE Systems, Inc., Bad Homburg, Germany) as previously described^4^. Each mouse was assessed for 72 h in the fed state to determine the metabolic rate. After an acclimation period of 4 days, heat generation, O_2_ consumption, and CO_2_ production were measured and the relationship between metabolic rate and body mass was normalized using metabolic body size (body weight^0.75^).

### Glucose and insulin tolerance tests

GTT was performed as described previously^62^. For NC-fed cohorts, male mice aged 24 weeks (*n*=5 each group; body weight, 30.93±1.01 g for WT and 29.74±1.26 g for Foxo1 KO^DAT^; *P*>0.45) were used. For HFD-fed cohorts, male mice fed HFD for 10 weeks (*n*=9 each group; body weight, 37.94±2.07 g for WT and and 32.74±1.31 g for FoxO1 KO^DAT^; *P*=0.04) were used. Glucose (1.5 g kg^−1^ body weight) was injected intraperitoneally.

For ITT, 24-week old NC-fed male mice (*n*=5 each group; body weight, 31.22±1.16 g for WT and 29.99±1.26 g for FoxO1 KO^DAT^; *P*>0.49) or male mice fed HFD for 10 weeks (body weight, 39.64±1.96 g for WT, *n*=8 and 33.86±1.55 g for FoxO1 KO^DAT^, *n*=11; *P*=0.03) were used. Insulin (0.9 U/kg; Eli Lilly and Company, IN, USA) was administered intraperitoneally.

### Body temperature

The rectal body temperature of FoxO1 KO^DAT^ and WT littermate mice at the age of 32 weeks for male and at the age of 30 weeks for female (*n*=5 each group) was measured in a room temperature environment at 9:00 hours by Thermalert TH-5 equipment (Physitemp Instruments, Inc., NJ, USA). Each mouse was measured three times and the average temperature was calculated.

### Sucrose preference test

Sucrose preference test was performed as described previously^63^. Briefly, 10-week-old male mice on chow diet (*n*=6 each group; body weight, 24.68±0.77 g for WT and 23.73±0.89 g for FoxO1 KO^DAT^; *P*>0.4) were used. Mice were housed in cages equipped with two drinking bottles, one containing filtered tap water and another one containing sucrose solution. The mice were first trained and acclimatized to the new environment for 3 days and then the sucrose preference test was performed using different concentrations of the sucrose solutions, 1, 2 and 5% (each concentration was tested for 3 days). The mice had free access to both bottles during the habituation and the experimental period. Fluid consumption was measured twice a day at 8:00 and 17:00 by weighing the bottles. To avoid side bias, the positions of the bottles were switched daily. On the last day of experiment period, mice were fasted overnight (with water *ad libitum*) and sucrose preference test was subsequently monitored with 2% sucrose. Sucrose preference was calculated as the percentage of the amount of sucrose solution consumed over total fluid consumption.

### Insulin and leptin measurement

Blood samples were collected from the tail-nicked blood drops for insulin and leptin measurements. Insulin and leptin levels were measured using ELISA kits (Morinaga Institute of Biological Science, Yokohama, Japan) in accordance with manufacturer's instructions.

### Measurement of norepinephrine (NE)

Plasma or serum samples were collected from mice in basal resting condition (daytime at 14:00 with food and water *ad libitum*). We measured NE using ELISA kit (LDN Labor Dianostika Nord GmbH & Co., KG, Nordhorn, Germany) as described elsewhere^64^.

### Measurement of dopamine (DA) content

DA levels from the midbrain containing VTA and SN samples were analysed by Biomedical Research Center, Asan Institute for Life Sciences (Asan Medical Center, Seoul, Korea) and were presented as pmole mg^−1^ of tissue. Briefly, dopamine hydrochloride and dopamine-1,1,2,2-d_4_ hydrochloride were purchased from Sigma-Aldrich (St Louis, MO, USA). Oasis wax cartridges were from Waters (Milford, MA, USA). All solvents including water were purchased from J.T. Baker (Center Valley, PA, USA).

The midbrain and SN samples were weighed and then homogenized using Tissue Lyzer (QIAGEN, Hilden, Germany) with 400 μl of methanol. The homogenate was incubated for 15 min at 4  °C. 200 μl of 1 μM dopamine-1,1,2,2-d_4_ hydrochloride, internal standard, was added into the sample after incubation, and mixed well. The sample was then centrifuged at 14,000 r.p.m. for 15 min. The supernatant was collected and equal volume of 1% formic acid was added. The sample was mixed well and ready for solid phase extraction. Oasis wax 3cc cartridge was conditioned with 1 ml of methanol and 0.5% formic acid, sequentially. The sample solution was loaded to the cartridge and incubated for 10 min. Then the cartridge was completely dried under vacuum. Finally, 1 ml of methanol was added for sample elution, and the eluant was dried under vacuum. The dried sample was stored at −20 °C until analysis and reconstituted with 20 μl of 50% methanol before Liquid Chromatography-Tandem Mass Spectrometry (LC-MS/MS) analysis.

Dopamine was analysed with LC-MS/MS equipped with 1290 HPLC (Agilent Technologies, CA, USA), Qtrap 5500 (AB Sciex, MA, USA), and a reverse phase column (Pursuit 5 C18 150 × 2 mm). 3 μl was injected into the LC-MS/MS system and ionized with turbo spray ionization source. 0.1% formic acid in H_2_O and 0.1% formic acid in methanol were used as mobile phase A and B, respectively. The separation gradient was as follows: hold at 10% B for 5 min, 10 to 70% B for 13 min, 70 to 90% B for 0.1 min, hold at 90% B for 8.9 min, 90 to 10% B for 0.1 min, then hold at 10% B for 2.9 min. LC flow was 200 μl min^−1^, and column temperature was kept at 23 °C. Multiple reaction monitoring was used in positive ion mode, and the extracted ion chromatogram (EIC) corresponding to the specific transition for each analyte was used for quantitation. The specific transitions (Q1/Q3) for dopamine and internal standard were 154.1/137.1 and 158.1/141.1, respectively. Data was analysed using Analyst 1.5.2 software.

### Cell culture

HEK293T and Neuro2A cells obtained from American Type Culture Collection (ATCC, VA, USA) were maintained in DMEM media containing 10% fetal bovine serum and 1% penicillin/streptomycin. Cells were transfected with Lipofectamine 2000 (Invitrogen, CA, USA) according to the manufacturer's instructions. For luciferase assay, cells were lysed 24 h after transfection. For mRNA and protein levels, cells were collected 48–96 h after transfection. All transfections were performed in triplicate.

### Vectors and siRNAs transfection

Vectors encoding myc-tagged FoxO1-WT, FoxO1-ADA (constitutively nuclear form with the mutations T24A, S253D and T316A) and FoxO1-DN (dominant-negative form lacking trans-activation domain with a deletion truncating after amino acid 265) and control vector, pCMV5, were used for FoxO1 overexpression experiments. FoxO1-specific siRNAs purchased from Bioneer (Bioneer Corp., Seoul, Korea, Cat.No. 1058762 and Cat.No. 1058758 for human FoxO1 siRNAs) and from Dharmacon (Dharmacon Inc., CO, USA, Cat.No. J-041127-08 and Cat.No. J-041127-07 for mouse FoxO1 siRNAs) were used to knockdown FoxO1.

### Immunoblotting

Cells were washed with 1 × PBS and lysed with RIPA buffer (150 mM NaCl, 50 mM Tris pH 8.0, 1% Triton X-100, 0.5% sodium deoxycholate and 0.1% SDS) containing protease and phosphatase inhibitors (Roche, Basel, Switzerland). For *in vivo* samples, the tissues were homogenized and lysed with RIPA buffer supplemented with protease and phosphatase inhibitors. The protein concentration was measured using Bio-Rad Protein Assay reagent (Bio-Rad Laboratories, CA, USA). Standard Western blotting was performed and the blots were visualized using the chemiluminescence UVP BioSpectrum 600 Imaging System (Fisher Scientific, MA, USA). Antibodies used for immunoblotting include FoxO1 (Cell Signaling, Cat.No. 2880, 1:2,000), FoxO1 (Abcam, Cat.No. ab39670, 1:2,000), p-Creb (Ser133) (Cell Signaling, Cat.No. 9196, 1:2,000), p38 MAPK (Cell Signaling, Cat.No. 9212, 1:2,000), p-p38 MAPK (Cell Signaling, Cat.No. 9211, 1:2,000), anti-TH (Milipore, Cat.No. AB152, 1:1,000), anti-TH phosphoSer40 (Milipore, Cat.No. AB5935, 1:1,000), anti-Dopamine D2 Receptor (Milipore, Cat.No. AB5084P, 1:1,000), c-myc (Roche, Cat.No. 11667149001, 1:1,000), GAPDH (Santa Cruz, Cat.No. sc-25778, 1:10,000), anti-Prolactin (Santa Cruz, Cat.No. sc-7805, 1:500), anti-PGC1α (Abcam, Cat.No. ab54481, 1:2,000), anti-Ucp1 (Abcam, Cat.No. ab10983, 1:10,000), anti-COMT (Abcam, Cat.No. ab126618, 1:1,000), anti-MAO-b (Abcam, Cat.No. ab125010, 1:2,000) and β-Actin (Abcam, Cat.No. ab6276, 1:10,000).

### Reverse transcription and quantitative real-time PCR

Total RNA was isolated using Ambion Trizol reagent (Life Technologies, CA, USA) and reverse transcribed to cDNA using High-Capacity cDNA Reverse Transcription Kits (Applied Biosystem, CA, USA) in accordance with the manufacturer's instructions. For reverse transcription PCR (RT-PCR), cDNAs were amplified using TaKaRa ExTaq (TaKaRa Bio Inc., Shiga, Japan) and the PCR products were analysed on 1.5% agarose gel. For quantitative real-time PCR, cDNA and primers were prepared with a *Power* SYBR Green PCR Master Mix (Applied Biosystem, Warrington, UK) according to the manufacturer's instructions.

For mitochondrial DNA content analysis, total DNA was extracted using DNAzol Reagent (Invitrogen, CA, USA) according to the manufacturer's instructions. Mitochondrial DNA was amplified using primers specific for the mitochondrial cytochrome c oxidase subunit 2 (*COX2*) gene and normalized to genomic DNA by amplification of the ribosomal protein s18 (*rps18*) nuclear gene^65^.

The primer sequences used for reverse transcription–PCR and quantitative real-time PCR can be found in the Supplementary Table 1.

### Immunohistochemistry

FoxO1 and TH immunoflourescence was performed on free-floating brain or adrenal cryosections using FoxO1-specific and TH-specific monoclonal antibodies (Milipore Technology, FoxO1, Cat.No. 04-1005 and TH, Cat.No. AB152). Briefly, mice were perfused with 4% neutral buffered formalin and the brains/adrenals were dissected and post-fixed in 4% formalin overnight. After cryoprotecting in 20% sucrose overnight, the brains/adrenals were cryo-sectioned into 20-μm slides in cryotome. Sections were washed in PBS 1X (pH 7.4) 3 times for 10 min each and were permeated in 0.5% Triton X-100 in PBS 1X (v/v) for 30 min. After blocking step with 3% goat serum prepared in PBS 1X containing 0.25% Triton X-100 (PBT) for one hour at RT, the sections were then incubated at 4 °C for 48 h with anti-FoxO1 primary antibody (1:200 dilution) and for 24 h with anti-TH primary antibody (1:1,000 dilution) in PBT-azide containing 3% (v/v) goat serum. The sections were then washed with PBS 1X 4 times for 10 min each and incubated for 2 h in Alexa Fluor 488 goat anti-rabbit antibody (Invitrogen, CA, USA, Cat.No. A21206, 1:200 dilution) in PBT containing 3% (v/v) goat serum. After washing 3 times in PBS 1 × 10 min each, the sections were mounted on glass slides and were visualized by confocal laser microscope.

UCP1 staining was performed on mounted paraffin-embedded sections of formalin-fixed BAT samples using UCP1 primary antibody (Abcam, Cat.No. ab10983, 1:1,000 dilution). After incubation with primary antibody at 4 °C for 24 h, the slides were washed 5 times with PBS 1X and then incubated with secondary antibody for 2 h using Vectastain Universal ABC kit (Vector Laboratories, CA, USA, Cat.No. PK-6200). After washing 3 times in PBS 1 × 10 min each, the slides were then stained and visible with 3,3'-diaminobenzidine (DAB)-peroxidase substrate solution (0.04% DAB and 0.01% H_2_O_2_).

### Generation of luciferase reporter constructs

The constructs of 5′proximal promoter regions of the mouse TH gene with or without potential binding sites of FoxO1 (TH_long, from −3,002 to −1, TH_medium, from −696 to −1 and TH_short, from −142 to −1) were amplified by PCR and cloned upstream of a luciferase reporter gene in pGL3-basic vector via *Kpn*I and *Nhe*I sites for TH_long and TH_short constructs and via *Xho*I and *Kpn*I sites for TH_medium construct. The PCR primers used include forward, 5′-GGGGGTACCGCTTCCCAGCTACTCC-3′ and reverse, 5′-CCCGCTAGCAAGCTGGTGGTCCC-3′ for TH_long construct; forward, 5′-GGGGGTACCGGGAGATGCCAAAGGC-3′ and reverse, 5′-CCCCTCGAGAAGCTGGTGGTCCCGA-3′ for TH_medium construct; forward, 5′-GGGGGTACCCCTCAGGCACAGCA-3′ and reverse, 5′-CCCGCTAGCAAGCTGGTGGTCCC-3′ for TH_short construct.

### Measurement of promoter activity

HEK293T cells were transfected with the following plasmids: FoxO1-WT (1,000 ng), FoxO1-ADA (1,000 ng), FoxO1-DN (1,000 ng), pCMV5 (1,000 ng), pGL3B (500 ng) or TH_long (500 ng), TH_medium (500 ng), TH_short (500 ng) and renilla (100 ng). After 24 h transfection, the activities of renilla and luciferase were determined using the Dual Luciferase Reporter Assay System (Promega, WI, USA). The luciferase activity was normalized to renilla activity. For FoxO1 knockdown experiments, cells were transfected with siRNA non-target control or FoxO1-specific siRNAs at final concentration of 100 nM together with pGL3B (500 ng) or TH_medium (500 ng) and renilla (100 ng) and luciferase assay was performed 48 h after transfection.

### Chromatin immunoprecipitation assay

We performed ChIP assays in isolated chromatin from cultured Neuro2A cells transfected with myc-tagged FoxO1-ADA and whole-brain samples from C57BL/6 mice using the following primers: proximal site, forward, 5′-GGAGTTCAGGATACTC-3′, reverse, 5′-GGATGTCTTCAAGTCCC-3′; middle site, forward, 5′-TTCTCTGAAGGGCTTGGGC-3′, reverse, 5′-CCCACTCACTCCCTGCAT-3′; distal site, forward, 5′-GGGACACAGCTAAAGTGCCC-3′, reverse, 5′-GCAACAGAAGCTAGGTG-3′; non-target site, forward, 5′-GGGTAATCCAGCATGGG-3′, reverse, 5′-AAGCTGGTGGTCCC-3′. The PCR conditions were 95 °C for 3 min and 40 cycles of denaturation at 95 °C for 30 s, annealing at 58 °C for 60 s and extension at 72 °C for 60 s for all primers. We used anti-FoxO1 rabbit polyclonal (Abcam, Cat.No. ab39670 and Chemicon, Cat.No. AB4130) or anti-c-myc mouse monoclonal (Roche, Cat.No. 11667149001) antibodies and protein A/G-coupled agarose beads (Thermo Scientific, CA, USA, Cat.No. 20422) to pull down FoxO1-chromatin complex.

### Statistics

The data are represented as mean±s.e.m. Statistical significance was determined by 2-tailed Student's *t*-test or analysis of variance. GraphPad PRISM version 5.0 was used for the statistical analyses, and *P*<0.05 was considered as a statistically significant difference.

### Data availability

The data and materials used for this study are available from the corresponding author on request.

## Additional information

**How to cite this article:** Doan, K. V. *et al*. FoxO1 in dopaminergic neurons regulates energy homoeostasis and targets tyrosine hydroxylase. *Nat. Commun.* 7:12733 doi: 10.1038/ncomms12733 (2016).

## Supplementary Material

Supplementary Information Supplementary Figures 1-7 and Supplementary Table 1.
