# Supplementary material for: FoxO1 in dopaminergic neurons regulates energy homeostasis and targets tyrosine hydroxylase
Source: Nat Commun. 2016 Sep 29;7:12733. doi: 10.1038/ncomms12733 (PMC5056402; doi:10.1038/ncomms12733)
Supplement: Supplementary Information — Supplementary Figures 1-7 and Supplementary Table 1. [file ncomms12733-s1.pdf]

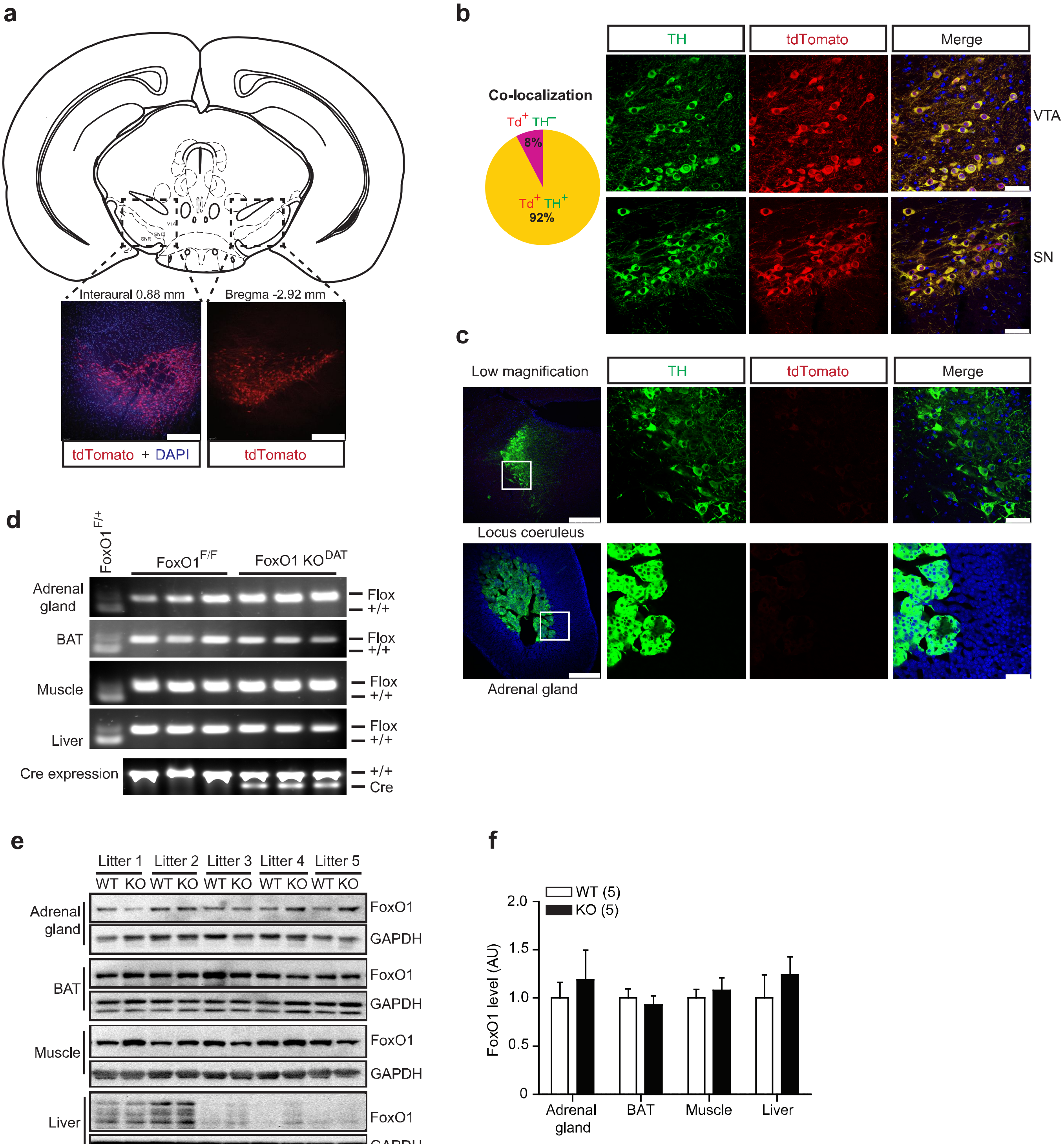
a
Interaural 0.88 mm
Bregma -2.92 mm
tdTomato + DAPI
tdTomato
b
Co-localization
Td+ TH−
8%
Td+ TH+
92%
TH
tdTomato
Merge
VTA
SN
c
Low magnification
TH
tdTomato
Merge
Locus coeruleus
Adrenal gland
d
FoxO1 F/+
FoxO1 F/F
FoxO1 KO DAT
Adrenal gland
BAT
Muscle
Liver
Cre expression
Flox
+/+
Cre
e
Litter 1
Litter 2
Litter 3
Litter 4
Litter 5
WT KO WT KO WT KO WT KO WT KO
Adrenal gland
BAT
Muscle
Liver
FoxO1
GAPDH
f
WT (5)
KO (5)
FoxO1 level (AU)
2.0
1.5
1.0
0.5
0
Adrenal gland
BAT
Muscle
Liver

Supplementary Figure 1. Validation of DA neuron-specific FoxO1 KO (FoxO1 KO^{DAT}) mice.

a, Cre expression in DA neurons of the midbrain of FoxO1 KO^{DAT} mice. Scale bar, 250 μm. SNC, substantia nigra-compact part. SNR, substantia nigra-reticular part. VTA, ventral tegmental area.

b, **c**, Co-localization between DAT-cre expression (red) and TH-immunoreactive cells (green) of the midbrain (**b**), locus coeruleus and adrenals (**c**). Scale bar, 50 μm (250 μm at low magnification). Percentage of co-localization of DAT-cre expression and TH-positive cells in the midbrain is 92.4 ± 2.5% (N=3).

d, Allele-specific PCR using indicated peripheral organs from $FoxO1^{F/+}$, $FoxO1^{F/F}$, and FoxO1 KO^{DAT} (DAT-cre; $FoxO1^{F/F}$).

e, Immunoblots for FoxO1 expression in indicated organs of WT and FoxO1 KO^{DAT} littermates.

f, Densitometry for FoxO1 protein levels from (**e**).

The results are expressed as mean ± SEM.

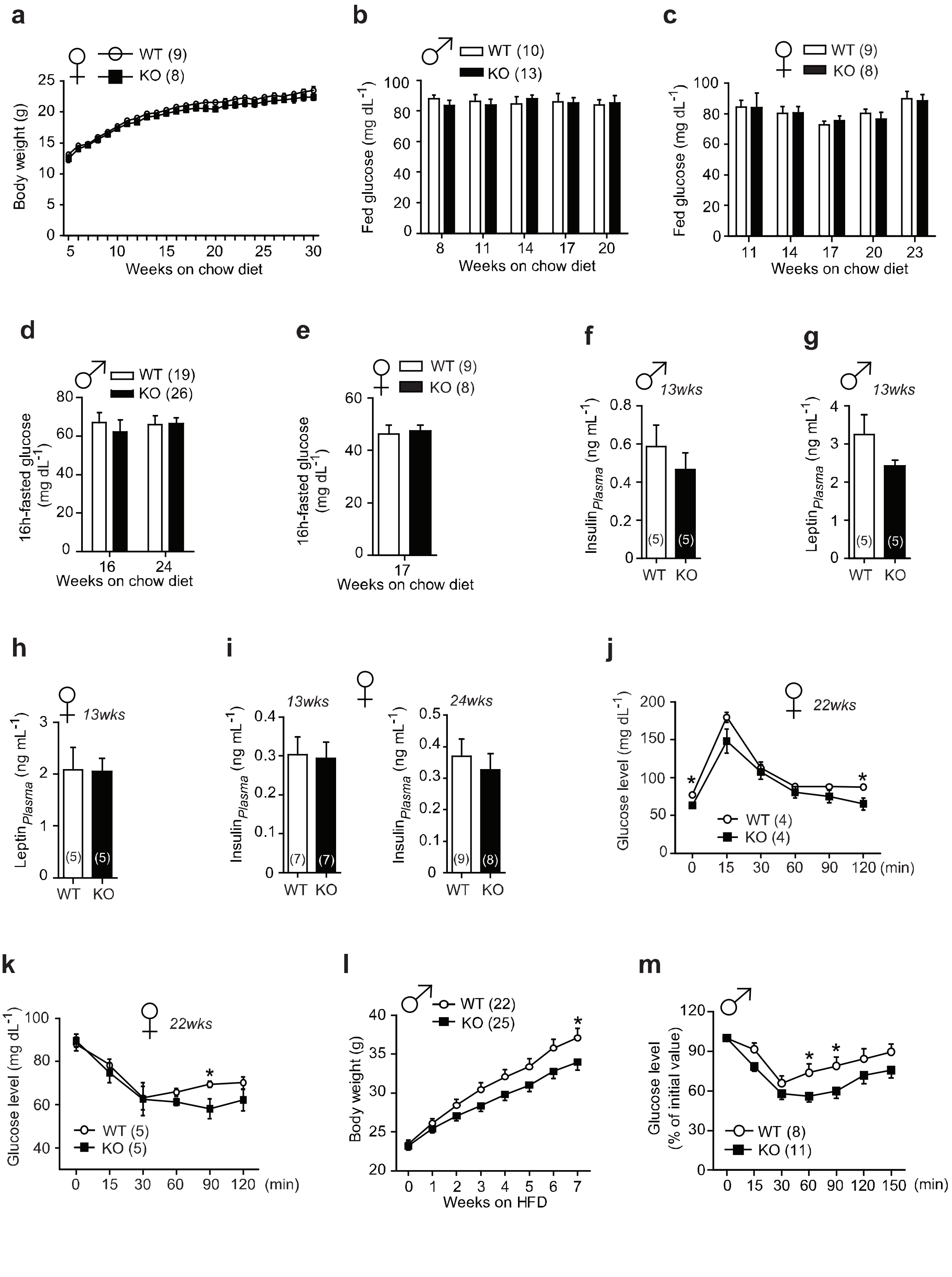

a
WT (9)
KO (8)
Body weight (g)
Weeks on chow diet
b
WT (10)
KO (13)
Fed glucose (mg dL-1)
Weeks on chow diet
c
WT (9)
KO (8)
Fed glucose (mg dL-1)
Weeks on chow diet
d
WT (19)
KO (26)
16h-fasted glucose (mg dL-1)
Weeks on chow diet
e
WT (9)
KO (8)
16h-fasted glucose (mg dL-1)
Weeks on chow diet
f
13wks
Insulin Plasma (ng mL-1)
WT KO
g
13wks
Leptin Plasma (ng mL-1)
WT KO
h
13wks
Leptin Plasma (ng mL-1)
WT KO
i
13wks
24wks
Insulin Plasma (ng mL-1)
WT KO
j
22wks
Glucose level (mg dL-1)
WT (4)
KO (4)
(min)
k
22wks
Glucose level (mg dL-1)
WT (5)
KO (5)
(min)
l
WT (22)
KO (25)
Body weight (g)
Weeks on HFD
m
WT (8)
KO (11)
Glucose level (% of initial value)
(min)

Supplementary Figure 2. Metabolic phenotypes of FoxO1 KO^{DAT} mice.

a, Body weight of WT and KO female mice on chow diet.

b, **c**, **d**, **e**, Fed (**b** and **c**) and fasted (**d** and **e**) blood glucose of WT and KO mice on chow diet.

f, **g**, Plasma insulin (**f**) and leptin (**g**) levels of WT and KO male mice on chow diet.

h, **i**, Plasma leptin (**h**) and insulin (**i**) levels of WT and KO female mice on chow diet.

j, **k**, GTT (**j**) and ITT (**k**) of WT and KO female mice on chow diet.

l, Weekly body weight after HFD (HFD started at 8 weeks old). Data were combined from 4 cohorts.

m, ITT results from Figure 3g normalized to initial glucose levels.

♂, male. ♀, female. The results are expressed as mean ± SEM (*P<0.05, Student's *t*-test for bar graphs and 2-way ANOVA for comparison of multiple time points in line graphs).

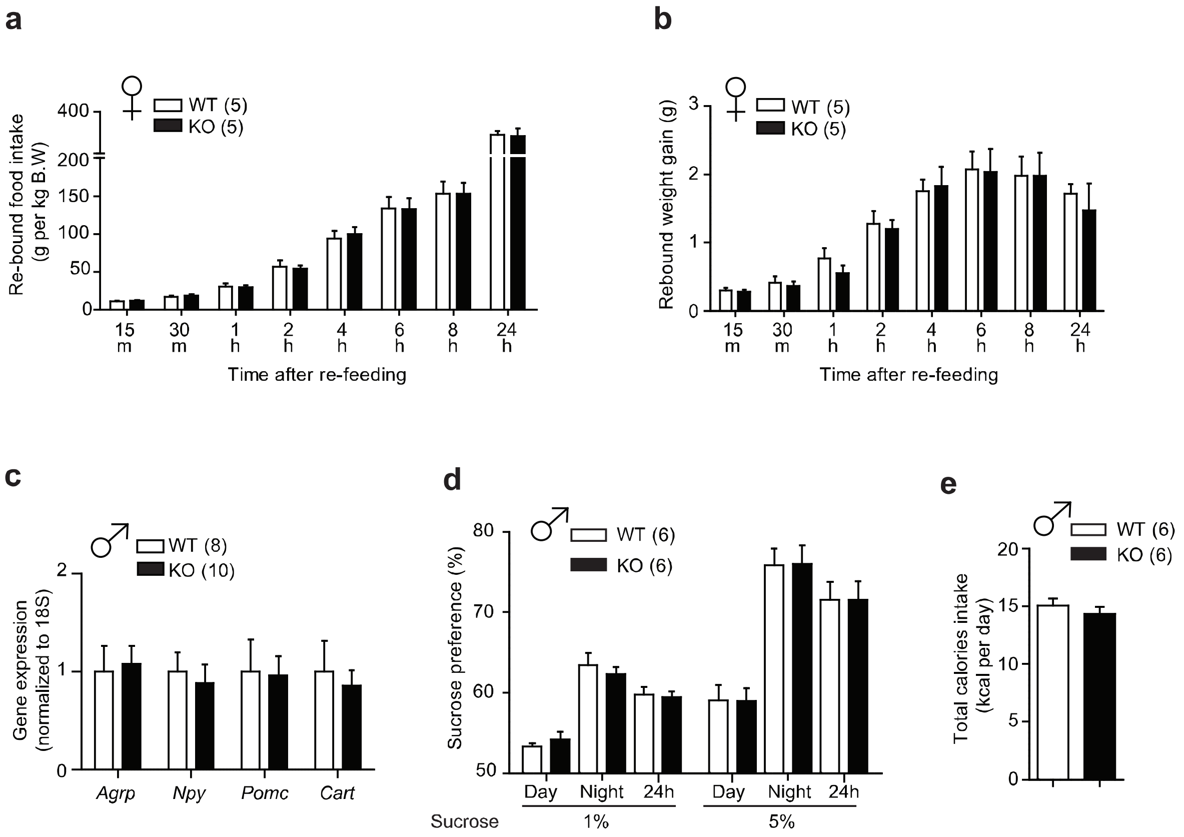

a
WT (5)
KO (5)
Re-bound food intake (g per kg B.W)
400
200
150
100
50
0
15 m
30 m
1 h
2 h
4 h
6 h
8 h
24 h
Time after re-feeding
b
WT (5)
KO (5)
Rebound weight gain (g)
3
2
1
0
15 m
30 m
1 h
2 h
4 h
6 h
8 h
24 h
Time after re-feeding
c
WT (8)
KO (10)
Gene expression (normalized to 18S)
2
1
0
Agrp
Npy
Pomc
Cart
d
WT (6)
KO (6)
Sucrose preference (%)
80
70
60
50
Day
Night
24h
Day
Night
24h
Sucrose
1%
5%
e
WT (6)
KO (6)
Total calories intake (kcal per day)
20
15
10
5
0

Supplementary Figure 3. Feeding behavior of FoxO1 KO^{DAT} mice.

a, **b**, Rebound food intake (**a**) and rebound weight gain (**b**) of overnight fasted WT and KO female mice after re-feeding with normal chow.

c, Hypothalamic gene expression from WT and KO male mice.

d, Percentage of sucrose preference of WT and KO male mice. Two different sucrose concentrations (1 and 5%) were used.

e, Total calories intake of WT and KO male mice recorded during sucrose preference test.

♂, male. ♀, female. The results are expressed as mean ± SEM.

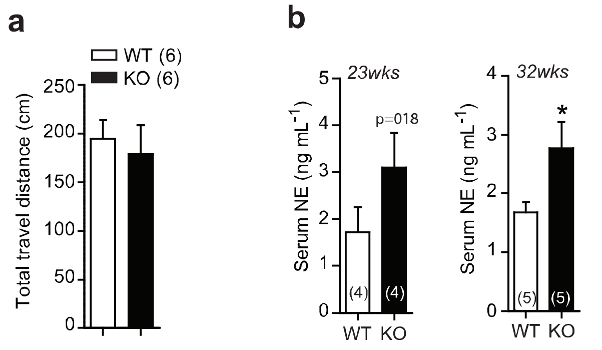

a
WT (6)
KO (6)
Total travel distance (cm)
250
200
150
100
50
0
b
23wks
Serum NE (ng mL-1)
p=018
(4)
(4)
WT
KO
32wks
*
(5)
(5)
WT
KO

Supplementary Figure 4. Increased energy expenditure and catecholamine levels in FoxO1 KO^{DAT} mice.

a, Locomotor activity of WT and KO male mice recorded from open field test.

b, Serum norepinephrine levels of WT and KO male mice fed normal chow at indicated age.

The results are expressed as mean ± SEM (*$P<0.05$, Student's *t*-test).

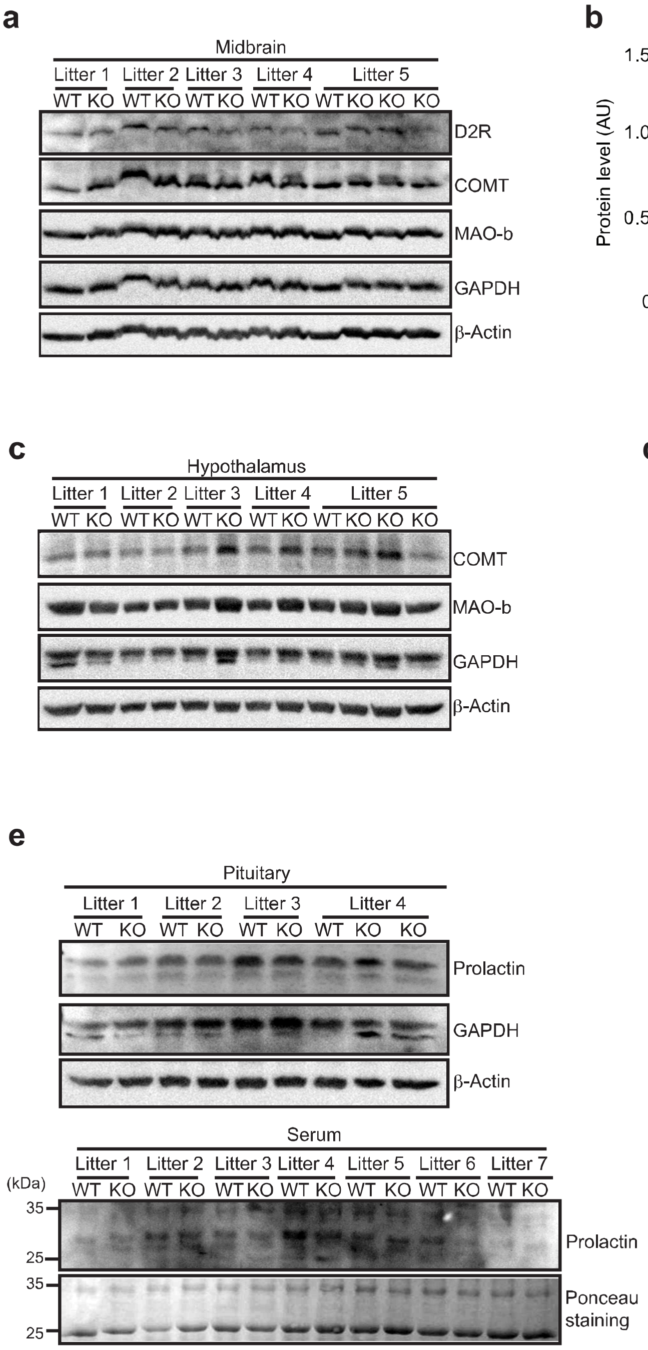

a
Midbrain
Litter 1
Litter 2
Litter 3
Litter 4
Litter 5
WT KO WT KO WT KO WT KO WT KO KO KO
D2R
COMT
MAO-b
GAPDH
β-Actin
b
WT (5)
KO (7)
Protein level (AU)
1.5
1.0
0.5
0
*
D2R
COMT
MAO-b
c
Hypothalamus
Litter 1
Litter 2
Litter 3
Litter 4
Litter 5
WT KO WT KO WT KO WT KO WT KO KO KO
COMT
MAO-b
GAPDH
β-Actin
d
WT (5)
KO (7)
Protein level (AU)
1.5
1.0
0.5
0
COMT
MAO-b
e
Pituitary
Litter 1
Litter 2
Litter 3
Litter 4
WT KO WT KO WT KO WT KO KO
Prolactin
GAPDH
β-Actin
Serum
Litter 1
Litter 2
Litter 3
Litter 4
Litter 5
Litter 6
Litter 7
WT KO WT KO WT KO WT KO WT KO WT KO WT KO
(kDa)
35
25
35
25
Prolactin
Ponceau staining
f
WT
KO
Prolactin level (AU)
1.5
1.0
0.5
0
(4)
(5)
(7)
(7)
Pituitary
Serum

Supplementary Figure 5. Expression of D2R, COMT, MAO-b and prolactin levels in FoxO1 KO^{DAT} mice.

a, **b**, Immunoblots (**a**) and relative protein levels (**b**) for D2R, COMT and MAO-b in midbrain samples of WT and KO mice.

c, **d**, Immunoblots (**c**) and relative protein levels (**d**) for COMT and MAO-b in hypothalamus of WT and KO mice.

e, **f**, Immunoblots (**e**) and relative prolactin levels (**f**) in the pituitary and serum samples of WT and KO mice.

The results are expressed as mean ± SEM (*P<0.05, Student's *t*-test).

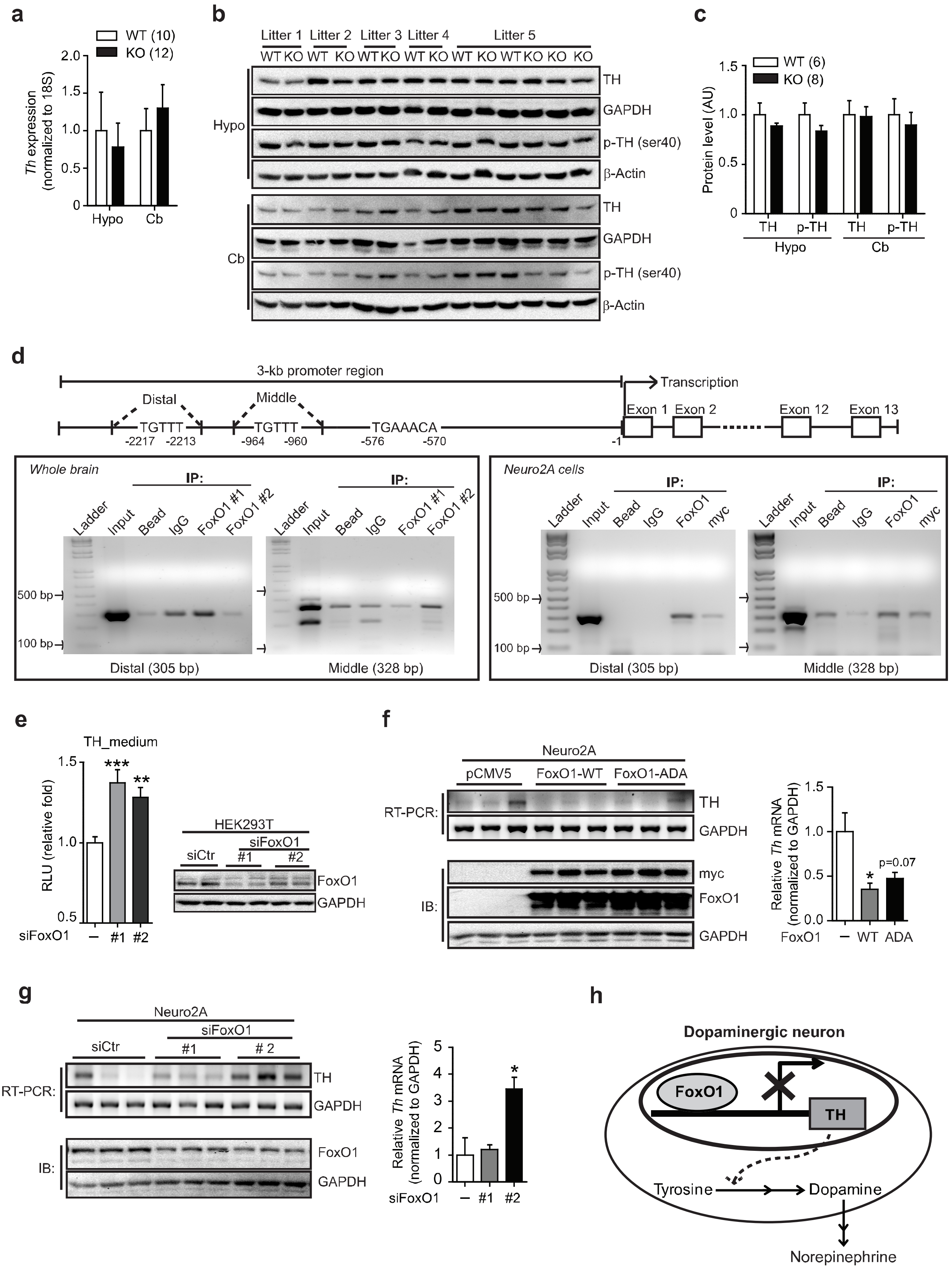
a
WT (10)
KO (12)
Th expression (normalized to 18S)
Hypo
Cb
b
Litter 1
Litter 2
Litter 3
Litter 4
Litter 5
Hypo
Cb
TH
GAPDH
p-TH (ser40)
β-Actin
c
WT (6)
KO (8)
Protein level (AU)
TH
p-TH
d
3-kb promoter region
Transcription
Distal
Middle
TGTTT
TGAAACA
Exon 1
Exon 2
Exon 12
Exon 13
Whole brain
Neuro2A cells
IP:
Ladder
Input
Bead
IgG
FoxO1 #1
FoxO1 #2
FoxO1
myc
500 bp
100 bp
Distal (305 bp)
Middle (328 bp)
e
TH_medium
RLU (relative fold)
siFoxO1
HEK293T
siCtr
f
Neuro2A
pCMV5
FoxO1-WT
FoxO1-ADA
RT-PCR:
IB:
Relative Th mRNA (normalized to GAPDH)
p=0.07
FoxO1
g
h
Dopaminergic neuron
FoxO1
TH
Tyrosine
Dopamine
Norepinephrine

Supplementary Figure 6. FoxO1 directly regulates tyrosine hydroxylase (TH) expression in DA neurons.

a, mRNA level of *Th* in the hypothalamus (Hypo) and cerebellum (Cb) of WT and KO littermate mice.

b, Immunoblots of TH and phosphorylated TH (p-TH) in the hypothalamus and cerebellum of WT and KO littermate mice.

c, Relative TH and p-TH protein levels from (**b**).

d, Top, schematic diagram for mouse TH promoter. Bottom, ChIP assays using whole-brain and Neuro2A cells transfected with myc-tagged FoxO1-ADA showing a non-specific binding of FoxO1 on the distal and middle regions of TH promoter.

e, Left, relative luciferase activity after FoxO1 knockdown by specific siRNAs (N=12). Right, immunoblots confirming the specific FoxO1 knockdown. The experiment was replicated two times.

f, Upper left, RT-PCR results showing the effect of FoxO1-WT and -ADA overexpression on the expression of endogenous *Th* mRNA in Neuro2A cells. Lower left, immunoblots confirming the FoxO1-WT and -ADA expression. Right, densitometry for *Th* mRNA levels from RT-PCR (N=3).

g, Upper left**,** RT-PCR results showing the effect of FoxO1 knockdown on the expression of endogenous *Th* mRNA in Neuro2A cells. Lower left, immunoblots confirming FoxO1 knockdown. Right, Densitometry for *Th* mRNA levels from RT-PCR (N=3).

h, Schematic diagram depicting FoxO1 regulation of TH expression and catecholamine synthesis in DA neurons.

The results are expressed as mean ± SEM (*P<0.05, **P<0.01, ***P<0.001, Student's *t*-test, one-way ANOVA for luciferase assays)

Supplementary Figure 7. Full-gel blots

Figure 1c

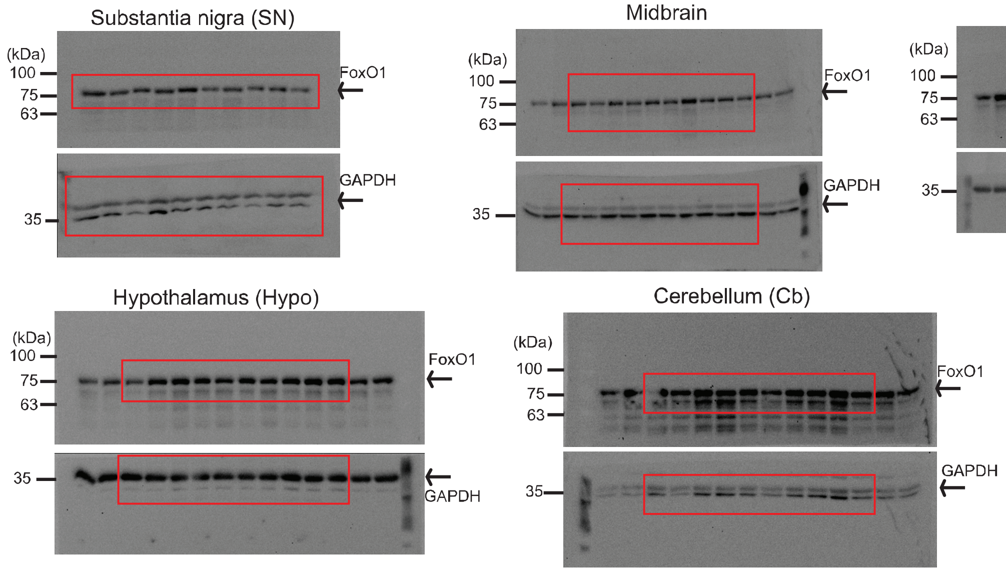

Figure 6d

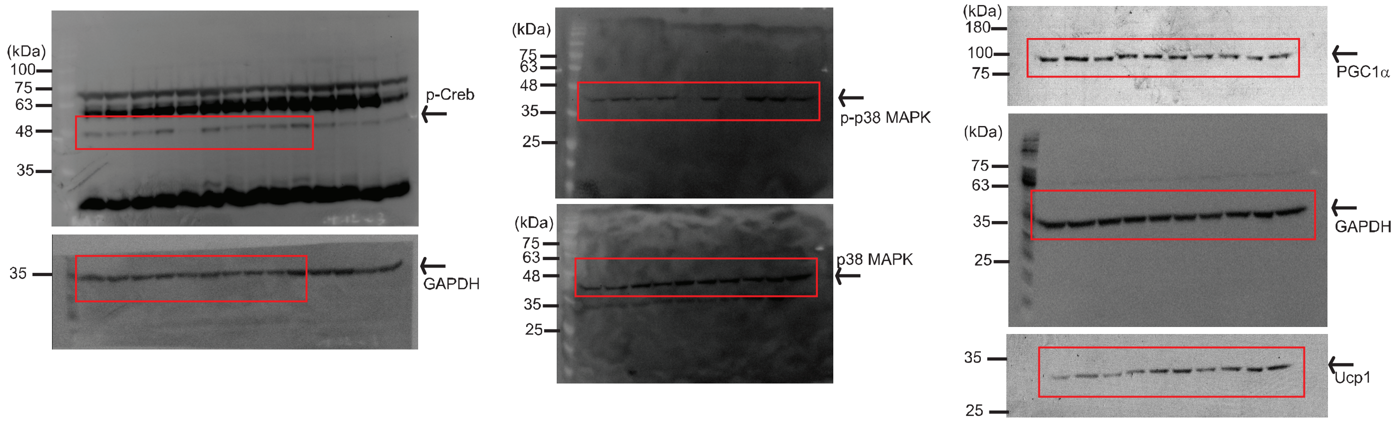

Figure 7b

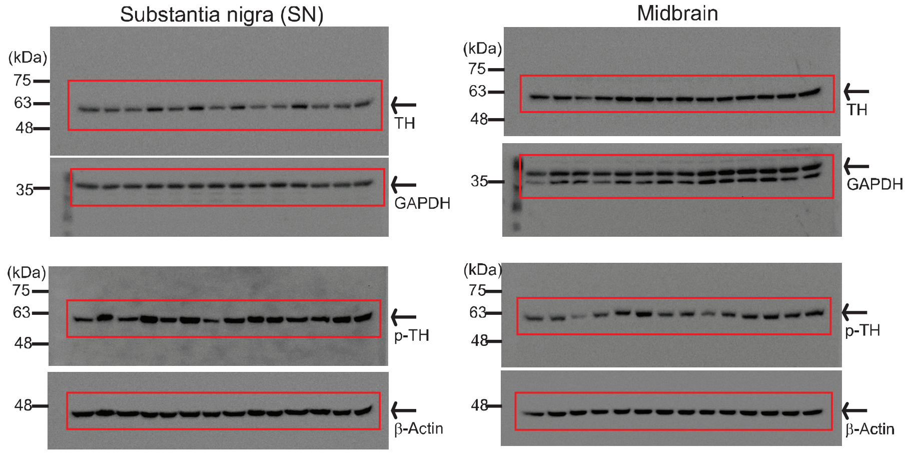

Figure 7e

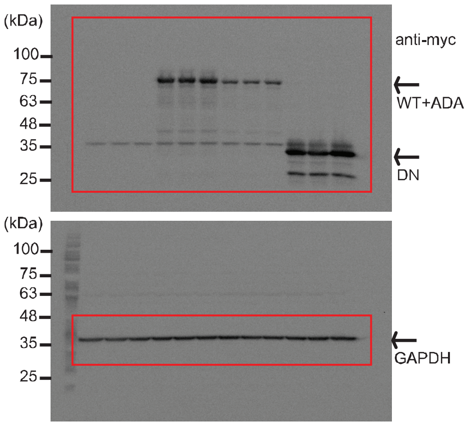

Supplementary Figure 7. Full-gel blots (continued)

Supplementary Fig. 1e

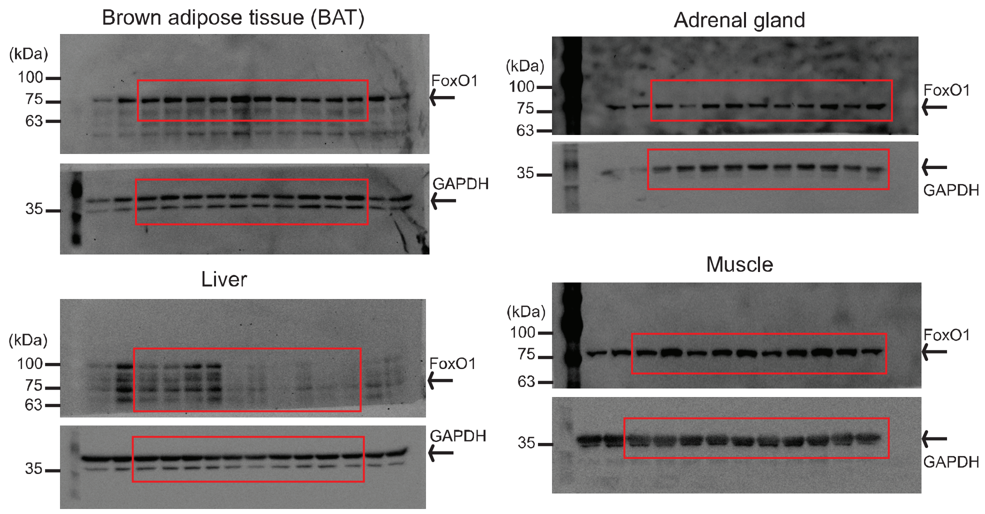

Supplementary Fig. 5a

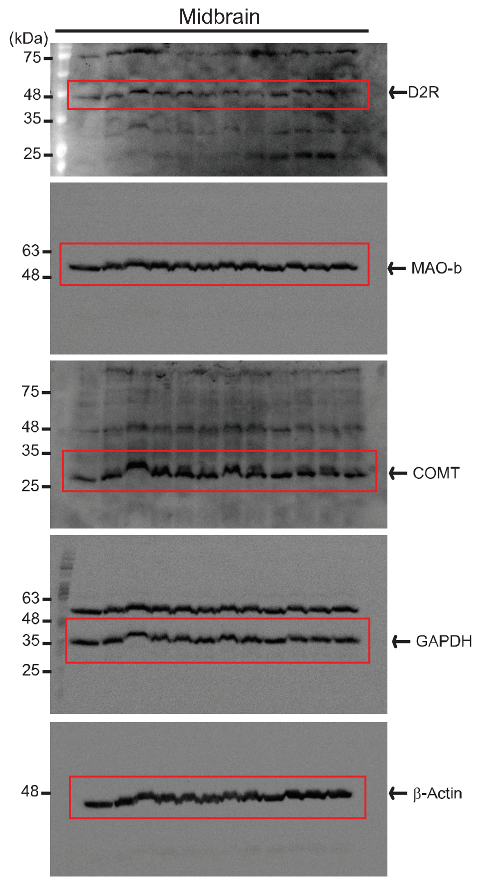

Supplementary Fig. 5c

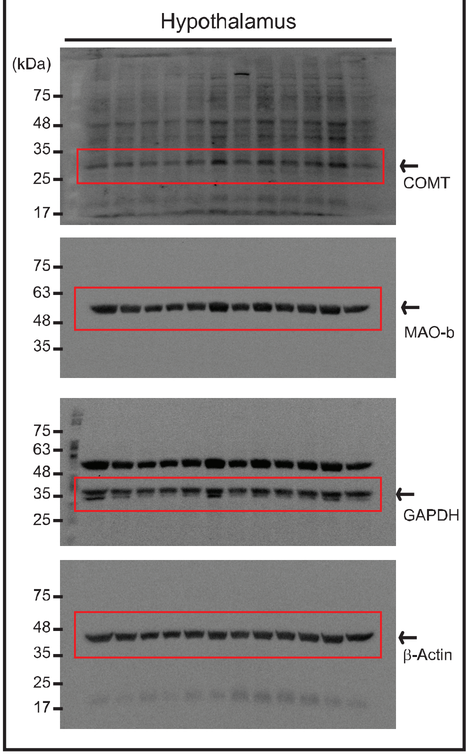

Supplementary Fig. 5e

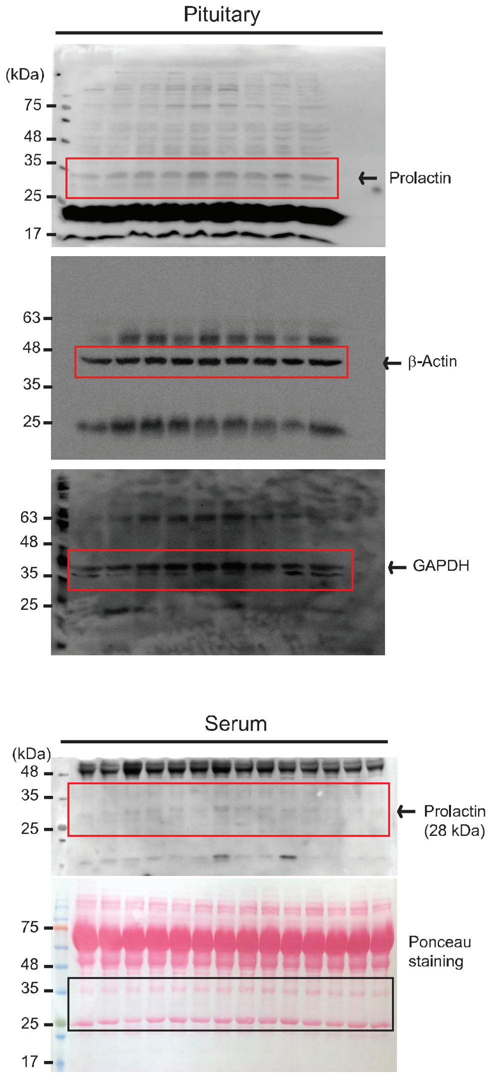

Supplementary Figure 7. Full-gel blots (continued)

Supplementary Fig. 6b

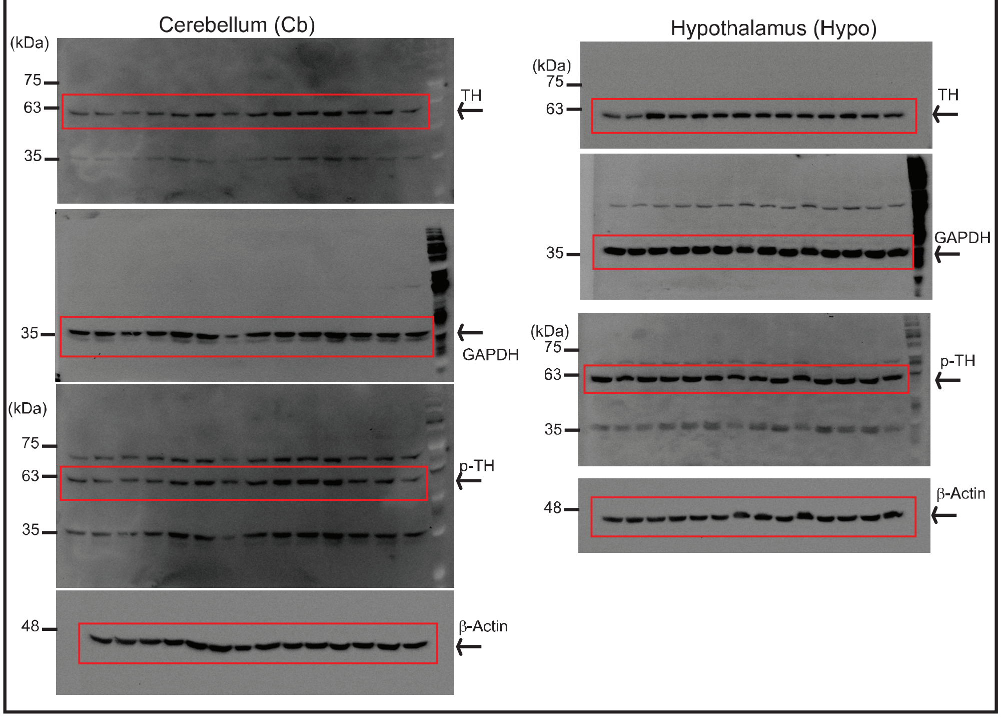

Supplementary Fig. 6e

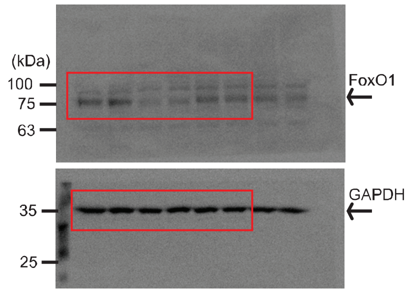

Supplementary Fig. 6g

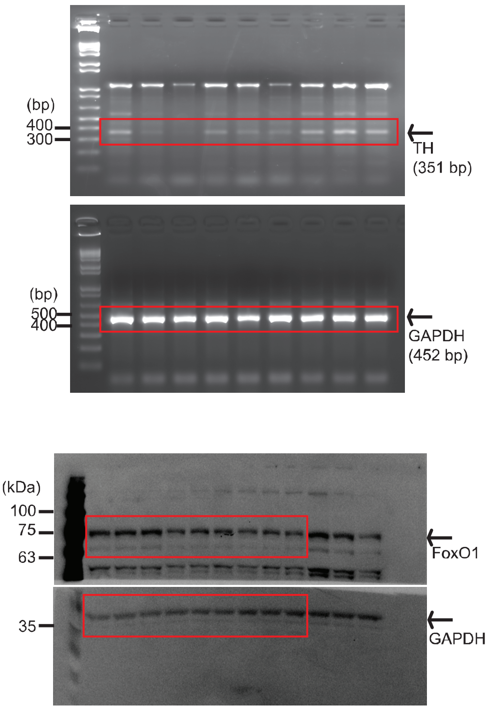

Supplementary Fig. 6f

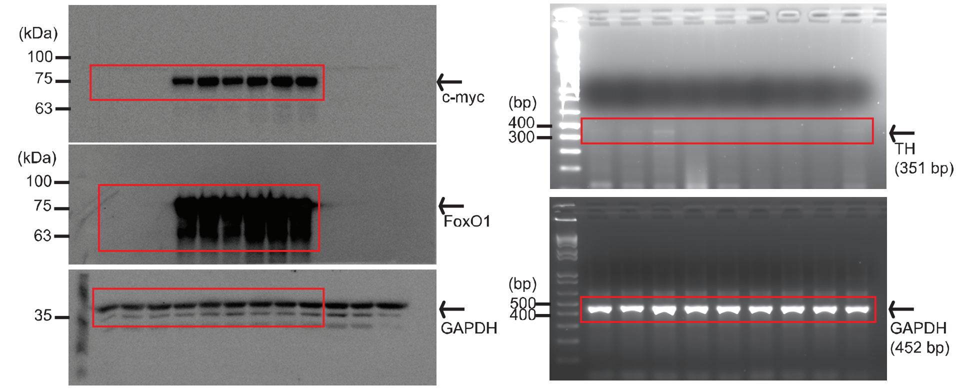

Supplementary Table 1

Sequences for mouse Q-PCR primers			
**Gene**	**Forward primer**	**Reverse primer**	**Product size (bp)**
18S	5’-AACCCGTTGAACCCCATT-3’	5’-CCATCCAATCGGTAGTAGCG-3’	149
Adrb3	5’- TGAAACAGCAGACAGGGACA -3’	5’- GGCGTCCTGTCTTGACACTC -3’	102
AgRP	5'-CGGCCACGAACCTCTGTAG-3'	5'-CTCATCCCCTGCCTTTGC-3'	65
CART	5'-AGAAGAAGTACGGCCAAGTC-3'	5'-GGACAGTCACACAGCTTCC-3'	91
NPY	5'-CTACTCCGCTCTGCGACACT-3'	5'-AGTGTCTCAGGGCTGGATCTC-3'	75
PGC1α	5’-AACCACACCCACAGGATCAGA-3’	5’-TCTTCGCTTTATTGCTCCATGA-3’	73
POMC	5'- CAGGTCCTGGAGTCCGAC -3'	5'- CATGAAGCCACCGTAACG -3'	102
TH	5’-TTGGCTGACCGCACATTT-3’	5’-GCCCCCAGAGATGCAAGT-3’	69
Ucp1	5’-GGCCCTTGTAAACAACAAAATAC-3’	5’-GGCAACAAGAGCTGACAGTAAAT-3’	67
Ucp3	5’- TTTCTGCGTCTGGGAGCTT-3’	5’- GGCCCTCTTCAGTTGCTCAT-3’	63
Rps18 (mtDNA analysis)	5’-TGTGTTAGGGGACTGGTGGACA-3’	5’-CATCACCCACTTACCCCCAAAA -3’	195
COX-2 (mtDNA analysis)	5’-ATAACCGAGTCGTTCTGCCAAT-3’	5’-TTTCAGAGCATTGGCCATAGAA-3’	180

Sequences for mouse RT-PCR primers			
**Gene**	**Forward primer**	**Reverse primer**	**Product size (bp)**
GAPDH	5’-ACCACAGTCCATGCCATCAC-3’	5’-TCCACCACCCTGTTGCTGTA-3’	452
TH	5’-TACCGAGAGGACAGGATTCC-3’	5’-TTTACACAGCCCAAACTCCA-3’	351
